# Accelerate Mass Transport of Proton and Carbon Sources by Super‐Hygroscopic and Porous Nanosheets for Continuous CO_2_‐To‐Ethylene Upgrade

**DOI:** 10.1002/advs.202502306

**Published:** 2025-05-14

**Authors:** Silong Dong, Guobin Wen, Xinyu Yang, Xiaowen Zhang, Shuxuan Liu, Haoyang Xiong, Yinyi Liu, Kai Zong, Hao Li, Yifan Li, Yi Cui, Bohua Ren, Xin Wang, Mingliang Jin, Zhongwei Chen

**Affiliations:** ^1^ South China Academy of Advanced Optoelectronics South China Normal University Guangzhou Guangdong 510006 China; ^2^ Institute of Carbon Neutrality Zhejiang Wanli University Ningbo 315100 China; ^3^ State Key Laboratory of Chem/Bio‐Sensing and Chemometrics College of Chemistry and Chemical Engineering National Supercomputer Centers in Changsha Hunan University Changsha Hunan 410082 China; ^4^ i‐lab Nano‐X Vacuum Interconnected Workstation Suzhou Institute of Nano‐Tech and Nano‐Bionics Chinese Academy of Sciences Suzhou Jiangsu 215123 China; ^5^ Department of Chemical Engineering Waterloo Institute for Nanotechnology University of Waterloo 200 University Avenue West Waterloo Ontario N2L 3G1 Canada; ^6^ State Key Laboratory of Catalysis‐Energy Power Battery and Systems Research Center Dalian Institute of Chemical Physics Chinese Academy of Sciences Dalian 116023 China

**Keywords:** capillary condensation, electrochemical CO_2_ reduction reaction, ethylene, H_2_O and CO_2_ channels, mass transport

## Abstract

Gas‐water/catalyst triple‐phase interface and the microenvironment play critical roles in the reaction kinetics and production rate of electrochemical carbon dioxide reduction reactions (CO_2_RR), which steer concerted proton‐electron transfer steps. Inspired by Tillandsia leaves, which efficiently capture H_2_O and CO_2_ from the air, copper nanosheets with dual‐functional channels are we designed: the superhygroscopic network enables capillary condensation, converting H_2_O(g) into H_2_O(l) to form H_2_O channels that ensure a stable supply of protons, while the CO_2_ channels formed by the microporous structure enhance the diffusion of CO_2_, thus enriching the carbon source. This synergistic design creates an optimal microenvironment for CO_2_ conversion by simultaneously delivering both protons and CO_2_ to the reaction interface. Time‐of‐flight secondary‐ion mass spectroscopy (TOF‐SIMS), X‐ray absorption spectroscopy (XAS) and multiphysics simulations further reveal the designed H_2_O and CO_2_ channels in the microenvironment to boost mass transports. Hence, the Faradaic efficiency (FE) for ethylene reaches up to 96% at ‐200 mA cm^−2^ with such localized triple‐phase interfaces, which simultaneously exhibits ultra‐high stability for over 170 h in the membrane electrode assembly (MEA) system. This strategy provides a construction methodology of H_2_O and CO_2_ channels for improving the selectivity and stability of electrochemical CO_2_ upgrades.

## Graphical abstract

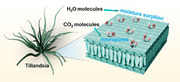

## 1 Introduction

Synthesis of ethylene via electrochemical carbon dioxide reduction reactions (CO_2_RR) demonstrates great potential for global carbon neutralization.^[^ ^1^, ^2^, ^3^ ^]^ However, the conversion rate is impeded by the chemical stability of CO_2_ molecules,^[^ ^4^ ^]^ which requires concerted proton‐electron transfer to break the C═O bond,^[^ ^5^, ^6^ ^]^ prompting it for further C─C coupling to form value‐added multicarbon (C_2+_) products. Hitherto, Copper materials have emerged as the most effective catalyst for converting CO_2_ into multi‐carbon hydrocarbons and oxygenates,^[^ ^7^, ^8^ ^]^ such as C_2_H_4_, C_2_H_6,_ and C_2_H_5_OH, with considerable activity.^[^ ^9^, ^10^, ^11^ ^]^ Although the adsorption capacity of catalysts for specific intermediates in diverse reaction pathways is relatively limited during the proton‐coupled electron transfer process, the limited mass transport of H_2_O and CO_2_ in localized triple‐phase interfaces of the copper catalyst further restricts the selectivity and production rate improvements with continuous operations.^[^ ^12^, ^13^, ^14^ ^]^

H_2_O molecules are a rich source of benign protons and play multiple roles in CO_2_RR, including solvent, proton donor, intermediate product conversion, and optimization of catalyst stability.^[^ ^15^, ^16^, ^17^, ^18^ ^]^ However, the limited diffusion rate of the H_2_O molecules affects the selectivity of CO_2_RR. Although researchers use a membrane electrode assembly (MEA) system to enhance ethylene selectivity, the limited availability of active catalyst surface sites within MEA systems and the excessive CO_2_ coverage on the catalyst surface may impede the carbon‐carbon coupling reaction, consequently reducing the ethylene conversion rate.^[^ ^19^, ^20^, ^21^ ^]^ Therefore, there remain substantial challenges in converting CO_2_ into C_2_H_4_ products.

**Scheme 1:**
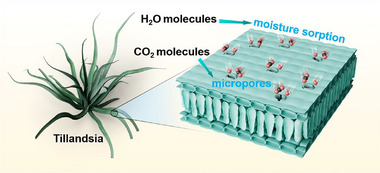
The conceptual diagram of the Tillandsia and a localized enlargement of its leaves.

The hygroscopicity of natural plants provides an instructive example for solving the problem of efficient H_2_O transport, such as Tillandsia species, as an interesting epiphyte, which have adaptively evolved hygroscopic leaves to replace degenerated roots, allowing them to absorb moisture from the atmosphere for the surviving.^[^ ^22^, ^23^ ^]^ Furthermore, there are a large number of micropores distributed on the surface of Tillandsia leaves, which allow gases and H_2_O molecules to enter the leaves (**Scheme** **1**). Therefore, the presence of these micropores not only expands the surface area of the leaves but also increases the permeability of gases and H_2_O molecules. These characteristics play a key role in the CO_2_RR process of MEA systems, which can increase diffusion rates of both CO_2_ and H_2_O, thus making the carbon sources and proton sources more abundant. This design boosts the production rate and selectivity of CO_2_RR, while reducing the polarization degree of the electrode, effectively inhibiting the competitive hydrogen production.^[^ ^24^ ^]^

Drawing inspiration from the hygroscopic Tillandsia, we strategically construct super‐hygroscopic microporous capillary condensed copper nanosheets (MPCC‐Cu) with H_2_O and CO_2_ channels. MPCC‐Cu is capable of condensing gaseous H_2_O molecules into a liquid state via capillary condensation, creating channels for the transport of protons, thereby enriching proton sources. The micropores on MPCC‐Cu provide channels for the diffusion transport of CO_2_ molecules, further leading to an increase in the diffusion rate of CO_2_ molecules, thereby facilitating carbon sources. Benefiting from the above advantages, MPCC‐Cu exhibits 93.6% selectivity for producing C_2+_ products in an H‐cell during CO_2_RR. The maximum FE_C2H4_ reaches 83.3% at −1.34 V vs RHE. Notably, in the MEA system, the maximum FE_C2H4_ reaches ≈96% at a current density of −200 mA cm^−2^. In addition, MPCC‐Cu also shows significant electrochemical stability at −200 mA cm^−2^ after 170 h of continuous operation. Consequently, this design greatly enhances the continuous CO_2_‐to‐ethylene upgrade in the MEA system.

## 2 Results and Discussion

### 2.1 Synthesis and Structural Characterizations of Catalysts

**Figure 1:**
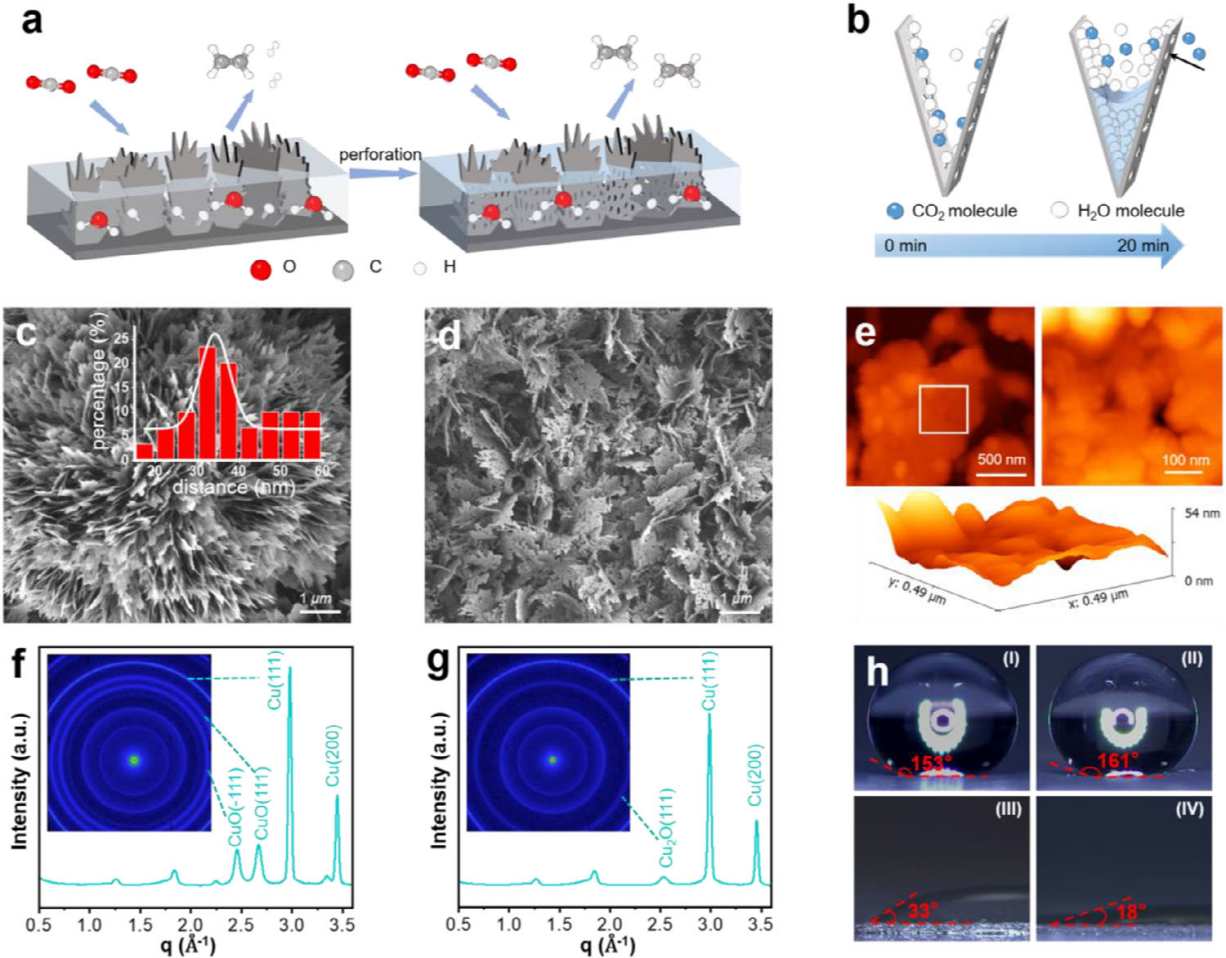
Structural characterizations of catalysts. a) The reconstruction process of converting CC‐Cu to MPCC‐Cu. b) Schematic diagram of the formation process of triple phase interface from 0 to 20 min with humid CO_2_ gas contacts MPCC‐Cu. c,d) SEM images of CC‐Cu (c) and MPCC‐Cu (d). The inset of (c) shows the distance distribution of adjacent nanosheets in the image in Figure S1 (Supporting Information). e) AFM image of MPCC‐Cu. The image on the right is the enlarged part of the white box, and the lower part is a 3D AFM image of the enlarged part. f,g) The WAXS 1D images of CC‐Cu (f) and MPCC‐Cu (g) correspond to 2D images in the illustrations. h) Contact angle test between carbon paper (I), ultrasonically treated carbon paper (II), CC‐Cu (III), and MPCC‐Cu (IV).

A way of activation was employed to reconstruct the crystal plane and obtain the required MPCC‐Cu (Supplementary information synthetic procedures), and conducted a series of characterizations of catalysts. In all the following discussions, the catalysts before and after activation and reconstruction processing are vividly referred to as capillary condensed copper nanosheets (CC‐Cu) and MPCC‐Cu, respectively. During the processing of the catalyst, ^*^H species generated by water splitting can combine with the surface of CC‐Cu to form metal hydride (CuH_x_), which is released into the electrolyte, resulting in the formation of small pores and gaps (**Figure** **1**a).^[^ ^25^ ^]^ This will further accelerate the mass transport of CO_2_ molecules and the diffusion rate of H_2_O molecules.^[^ ^9^ ^]^ Simultaneously, CC‐Cu exhibits a high propensity for capillary condensation phenomena, attributed to its hygroscopicity and the close proximity of the nanosheets, which are less than 100 nm apart.^[^ ^26^, ^27^, ^28^ ^]^ The distance between two adjacent nanosheets was analyzed, and most distances were ≈35 nm (Figure 1c inset; Figure S1, Supporting Information). Previous research has reported that when the material pores are cylindrical, the concave liquid surface formed by capillary condensation of H_2_O (g) is a spherical surface.^[^ ^29^, ^30^, ^31^ ^]^ As per the Kelvin equation, the relative pressure P/P^0^ and the spherical radius *r* are related as follows (Equation 1):^[^ ^32^ ^]^

(1) $$ln\;\frac{P}{P^{0}}=\frac{2\gamma M}{\rho RT}\;\cdot \frac{1}{r}$$

where P/P^0^ is the relative pressure, γ represents the surface tension, M denotes the molar mass of H_2_O, ρ is the density of liquid H_2_O, R denotes the gas constant; T and r respectively stand for the absolute temperature and radius of curvature of the meniscus. When measuring the distance between two adjacent nanosheets, we assume that they are parallel and the distance is *d*, then we can get *d*  =  2*r*cos θ, where *r* is the meniscus curvature radius and *θ* is the H_2_O and catalyst contact angle of the surface (0° < *θ*<90°). *d* is a constant value, so *r* is proportional to *θ*. When the liquid surface is concave, both sides of Equation (1) become negative. As P decreases, the absolute value of the left side of Equation (1) increases. Consequently, as P diminishes, *r* also diminishes. In other words, when *r* is smaller, capillary condensation can be formed under smaller pressure.

When MPCC‐Cu is assembled in the MEA system, the concentration of H_2_O molecules can be reduced and its hygroscopicity can be fully utilized to construct the triple‐phase interfaces (Figure 1b; Figure S2, Supporting Information). The interfaces offer more reaction sites during CO_2_RR, thereby facilitating the progress of the reaction. On the interfaces, CO_2_ molecules and H_2_O molecules can undergo chemical reactions with the active sites on the catalyst surface, resulting in the generation of CO_2_ conversion products.

**Figure 2:**
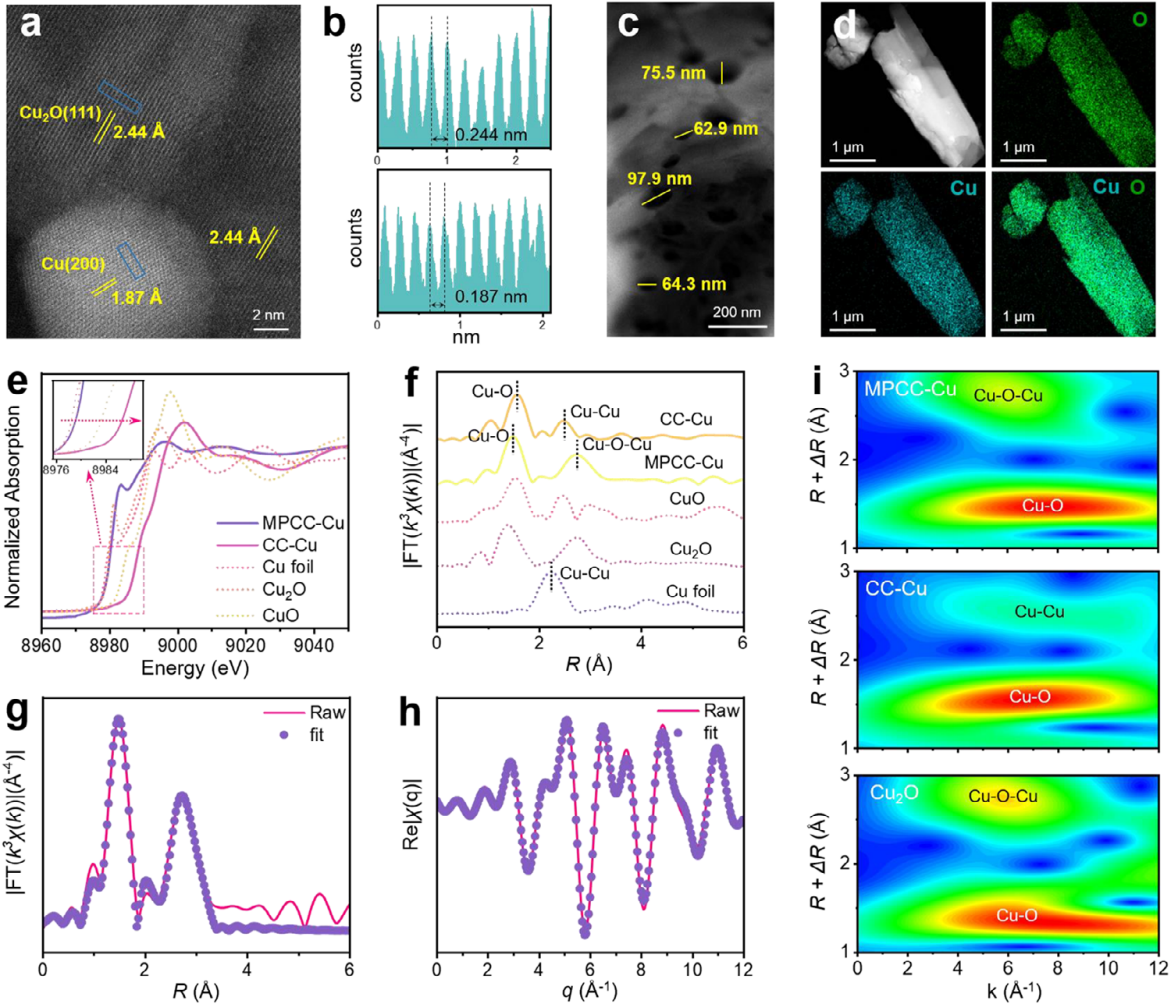
Microscopy characterizations and spectroscopy characterizations of catalysts. a–c) HRTEM image of MPCC‐Cu. Line‐scanning intensity profile b) of the lattice stripe spacing of the blue rectangular part in (a). d) HAADF‐STEM image and EDS element mapping of MPCC‐Cu. e) Normalized intensity of Cu K‐edge XANES spectra for CuO, Cu_2_O, Cu foil, CC‐Cu and MPCC‐Cu. f) Fourier‐transform k^3^‐weighted EXAFS spectra in R space for MPCC‐Cu and CC‐Cu. g,h) EXAFS fitting curves in R space (g) and q space (h) of MPCC‐Cu. i) Wavelet‐transformed k^3^‐weighted EXAFS spectra of MPCC‐Cu, CC‐Cu and Cu_2_O.

Scanning electron microscopy (SEM) was employed to examine CC‐Cu, revealing the presence of nanosheets (Figure 1c; Figure S3, Supporting Information). Atomic force microscopy (AFM) was used to determine the thickness of the nanosheet, which was found to be ≈10 nm (Figure S4, Supporting Information). Numerous micropores and cracks appeared on the surface of MPCC‐Cu nanosheets obtained after activation (Figure 1d; Figure S5, Supporting Information). AFM and HRTEM images further support this observation (Figures 1e and 2c; Figure S6, Supporting Information). X‐ray diffraction (XRD) pattern of CC‐Cu revealed the presence of both CuO and Cu phases (Figure S7, Supporting Information). Diffraction peaks at 32.7°, 35.7°, and 38.9° correspond to the (110), (‐111), and (111) planes of CuO, respectively (JCPDS PDF#74‐1021). Furthermore, diffraction peaks at 43.3° and 50.5° were observed corresponding to the (111) and (200) planes of Cu, respectively (JCPDS PDF#85‐1326). After activation, both Cu_2_O and Cu phases were present in MPCC‐Cu, with different diffraction peaks corresponding to Cu_2_O (JCPDS PDF#74‐1230) and Cu (JCPDS PDF#85‐1326). The (111) plane of Cu_2_O was observed at 36.6°, while (111) and (200) planes of Cu were also detected, in addition to the peaks of Cu_2_O. Wide‐angle X‐ray scattering (WAXS) further characterized the crystal planes of the catalyst. 1D curves (Figure 1f,g) and corresponding 2D patterns (insets) were obtained. The results are consistent with XRD patterns (Figure S7, Supporting Information).

We examined the hydrophilic and hydrophobic properties of the hydrophobic carbon paper (YLS‐30T, Toray, Japan), carbon paper after ultrasonic treatment, CC‐Cu, and MPCC‐Cu by measuring the contact angle. The contact angle of hydrophobic carbon paper is 153°. Its contact angle becomes larger after ultrasonic treatment. In comparison, CC‐Cu had a contact angle of 33° and MPCC‐Cu had a contact angle of 18° (Figure 1h), indicating that the catalyst we obtained was hydrophilic. Combining Eq. 1, we speculate that the reason why MPCC‐Cu is more hydrophilic than CC‐Cu is that many micropores and gaps appear on the surface of the catalyst after reconstruction, making it easier for the conditions for capillary condensation to occur.^[^ ^26^ ^]^

High‐resolution transmission electron microscopy (HRTEM) revealed that the lattice spacings of MPCC‐Cu were 0.244 nm and 0.205 nm, corresponding to the (111) plane of Cu_2_O and the (111) plane of Cu respectively (**Figure** **2**a,b). And the lattice spacings of CC‐Cu were 0.235 nm and 0.203 nm, corresponding to the (111) plane of CuO and the (111) plane of Cu, respectively (Figure S8, Supporting Information). The results are consistent with XRD patterns. This shows that their active interfaces are different. The active interface of MPCC‐Cu is Cu^+^/Cu^0^, while the active interface of CC‐Cu is Cu^2+^/Cu^+^. Through a comparative analysis of high‐angle annular dark‐field scanning transmission electron microscopy (HAADF‐STEM) and the corresponding energy‐dispersive X‐ray spectroscopy (EDS) images of MPCC‐Cu and CC‐Cu, it was observed that Cu (blue) and O (green) exhibited similar morphology and uniform distribution (Figure 2d; Figures S9 and S10, Supporting Information). These noteworthy findings provide valuable insights into the structural and compositional characterization of MPCC‐Cu and CC‐Cu, enhancing our understanding of their catalytic performance and potential applications.

### 2.2 Coordination Structures of MPCC‐Cu and CC‐Cu

In order to understand the chemical state, electronic structure, and coordination environment of Cu species in MPCC‐Cu and CC‐Cu, X‐ray absorption near‐edge structure (XANES) and extended X‐ray absorption fine structure (EXAFS) analyses were performed. The Cu foil, Cu_2_O, and CuO were also tested as a comparison to MPCC‐Cu and CC‐Cu. The main peak of ∼8980 eV in the Cu‐K‐edge XANES was due to the electronic transition from 1s to the unoccupied 4p orbit (Figure 2e).^[^ ^33^ ^]^ The white line peaks of MPCC‐Cu and Cu_2_O were similar, suggesting that the Cu valence state in MPCC‐Cu was close to Cu^+^. The white line peak of CC‐Cu showed a positive shift compared to CuO, indicating that the predominant valence state of Cu in CC‐Cu was Cu^2+^. This suggested that the Cu valence state is more stable within the structure of MPCC‐Cu. The Fourier transform‐EXAFS (FT‐EXAFS) curves of MPCC‐Cu and CC‐Cu both exhibited two main coordination peaks (Figure 2f). The initial coordination peak of MPCC‐Cu aligned with the Cu─O bond at 1.50 Å, while the subsequent coordination peak corresponded to the Cu─O─Cu bond at 2.75 Å. The primary coordination peak of CC‐Cu aligned with the Cu─O bond at 1.58 Å, while the secondary coordination peak corresponded to the Cu─Cu bond at 2.48 Å, akin to CuO. Notably, compared with Cu_2_O, the Cu─O bond length of the first shell layer of MPCC‐Cu was slightly shifted higher. Combined with EXAFS fitting (Figure 2g,h; Figure S11 and Table S2, Supporting Information), the coordination number of the Cu─O path was reduced from 2.2 to 0.7. We speculate that the coexistence of micropores and gaps combined with interfacial interactions between Cu and Cu_2_O phases in MPCC‐Cu leads to this result.^[^ ^34^, ^35^ ^]^ In the EXAFS wavelet transform spectrum of MPCC‐Cu, two intensity maxima were identified at ≈8 and 6 Å^−1^, attributed to the Cu─O and Cu─O─Cu bonds, respectively, resembling Cu_2_O. Similarly, in the EXAFS wavelet transform spectrum of CC‐Cu, two peaks of maximum strength were observed at ≈7 and 8 Å^−1^, corresponding to the Cu─O and Cu─Cu bonds, respectively, akin to CuO (Figure 2i; Figure S12, Supporting Information).

**Figure 3:**
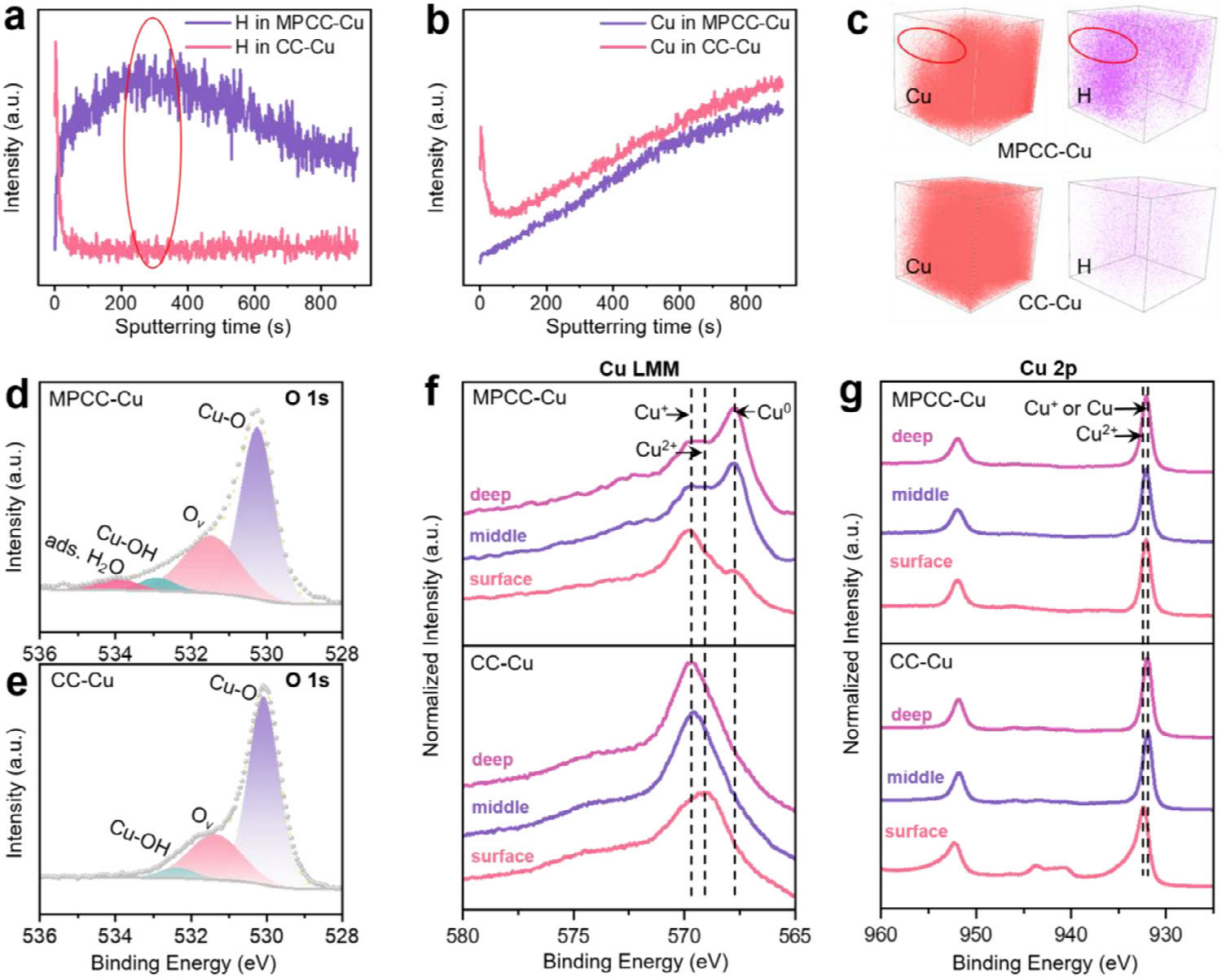
TOF‐SIMS and XPS characterizations at different depths. a,b) TOF‐SIMS depth profiles of MPCC‐Cu and CC‐Cu. Fragment ions of H (a) and Cu (b). c) TOF‐SIMS 3D spatial distribution of H and Cu elements for MPCC‐Cu and CC‐Cu. (d,e) O 1s XPS spectra of MPCC‐Cu d) and CC‐Cu e). f,g) Cu LMM (f) and Cu 2p (g) XPS spectra at different depths of MPCC‐Cu and CC‐Cu.

Time‐of‐flight secondary ion mass spectroscopy (TOF‐SIMS) can visualize the distribution of elements, thus allowing for further in‐depth research on the compositional differences and evolution of CC‐Cu and MPCC‐Cu. **Figure** **3**a,b show TOF‐SIMS depth profiles of H and Cu from CC‐Cu and MPCC‐Cu, respectively. Figure 3c is their corresponding 3D spatial distribution images. Combining the corresponding differences within the red circle can be seen that the content of Cu element in MPCC‐Cu is lower than in CC‐Cu, so we speculate that it may be related to the micropores on MPCC‐Cu. Meanwhile, H content in MPCC‐Cu is higher than in CC‐Cu. This indicates that MPCC‐Cu can better condense H_2_O molecules and enhance proton transport. To ascertain the source of H, X‐ray photoelectron spectroscopy (XPS) analyses were performed (Figure 3d,e), the O 1s revealed a discernible peak at 534.02 eV in the spectrum of MPCC‐Cu, the corresponding peak of adsorbed H_2_O,^[^ ^36^ ^]^ whereas such a peak was absent in the spectrum of CC‐Cu. This definitive observation corroborates the accuracy of our conclusion.

To better explain the changes in the electronic structure and chemical state of the catalysts, XPS analysis was carried out at various etching depths. The Cu LMM XPS region of CC‐Cu and MPCC‐Cu is shown in Figure 3f. The peaks observed at 567.7, 568.5, and 569.6 eV correspond to Cu^0^, Cu^2+,^ and Cu^+^ species, respectively.^[^ ^37^, ^38^, ^39^ ^]^ Obviously, CC‐Cu exhibits a surface mainly enriched in Cu^2+^, aligning seamlessly with the findings from the XANES result; However, as etching depth increases, the surface composition transitions to Cu^+^. MPCC‐Cu shows a surface predominantly enriched in Cu^+^, also in agreement with the results of the XANES result. As etching depth increases, Cu^+^ species intensity decreases while Cu^0^ species intensity increases. Additionally, satellite peaks of Cu^2+^ were detected in the Cu 2p XPS spectrum of CC‐Cu in the energy range of 940 to 945 eV (Figure 3g). Oxidation of copper nanosheets in air has been reported.^[^ ^39^, ^40^ ^]^ Therefore, we speculate that the Cu^+^ catalytic site in CC‐Cu is very active and is easily oxidized to Cu^2+^ by air. Moreover, significant Cu^+^ peaks were evident on the surface of MPCC‐Cu in both the Cu LMM and Cu 2p XPS spectra. These findings imply that the electrochemical reaction leads to the reduction of Cu^2+^ to Cu^+^ and Cu^0^, with some internal Cu^+^ being further reduced to Cu^0^. The presence of Cu^+^ promotes the C‐C coupling mechanism, thereby enhancing ethylene selectivity.

### 2.3 CO_2_RR Performance of MPCC‐Cu

The CO_2_RR performance of MPCC‐Cu was assessed through chronoamperometry measurements in a gas‐tight H‐cell, with 0.5 M KHCO_3_ saturated with CO_2_ as the electrolyte. The resulting electrolytic products were analyzed using online gas chromatography (GC) and ^1^H nuclear magnetic resonance (NMR) spectroscopy. All potentials were referenced to a reversible hydrogen electrode (RHE) unless otherwise noted. The gas phase products included C_2_H_4_, C_2_H_6_, H_2_, CO, and CH_4_. Since the majority of the CO_2_RR products in the catalyst were observed in the gas phase, with only a minor quantity of liquid products, our focus in the main text is solely on the gas phase products.

**Figure 4:**
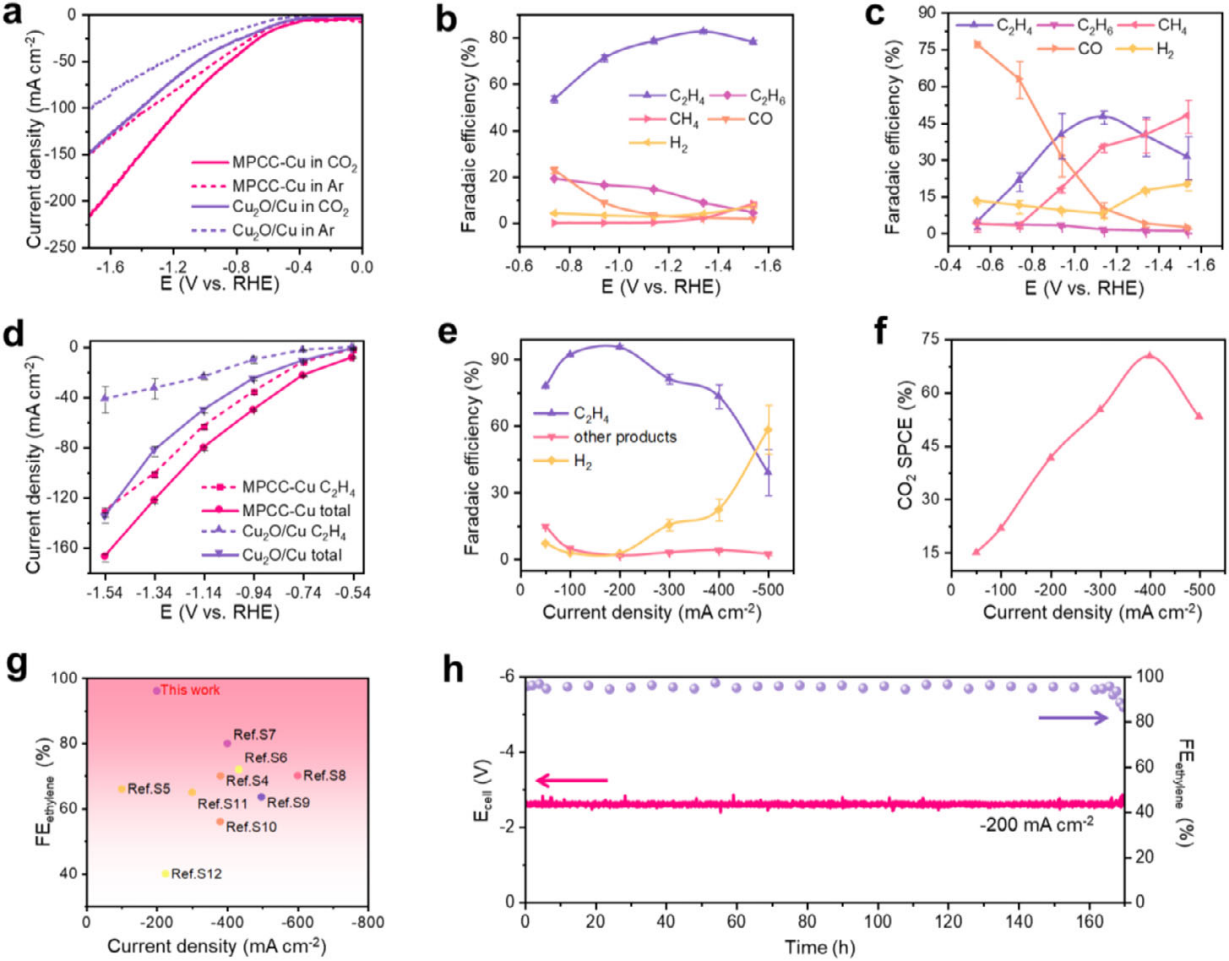
Electrochemical CO_2_RR performance of MPCC‐Cu and Cu_2_O/Cu catalysts. a) LSV curves of MPCC‐Cu and Cu_2_O/Cu catalyst in Ar‐saturated or CO_2_‐saturated electrolytes range from 0 to ‐1.74 V, with a scanning rate of 0.1 V s^−1^. b,c) FE of various products of MPCC‐Cu (b) and Cu_2_O/Cu catalyst (c) with potentials ranging from ‐0.64 to ‐1.54 V in the H‐cell. d) Current density and ethylene partial current density of MPCC‐Cu and Cu_2_O/Cu catalyst in the H‐cell. e) FE changes of MPCC‐Cu products under different current densities in the MEA system. f) CO_2_ SPCE at different current densities in the MEA system. g) Comparison of FE_C2H4_ for MPCC‐Cu with the reports. h) Stability test of MPCC‐Cu within 170 h at −200 mA cm^−2^ in the MEA system.

As a comparison, we synthesized a Cu_2_O/Cu catalyst with both Cu_2_O and Cu phases (Figures S13–S15, Supporting Information). The linear sweep voltammetry (LSV) curve highlights that MPCC‐Cu exhibits superior activity compared to the Cu_2_O/Cu catalyst, displaying a lower initial potential and higher current density (**Figure** **4**a). As the potential increases, both catalysts exhibit an increase in FE_C2H4_ and a decrease in FE_CO_, indicating the formation of ethylene through CO coupling at a high overpotential in the H‐cell (Figure 4b; Figure S16, Supporting Information). Within the wide potential range of −0.94 to −1.54 V, the FE_C2H4_ on the MPCC‐Cu maintains >70%, much higher than that on Cu_2_O/Cu (Figure 4c). At a potential of −1.14 V, the FE of C_2+_ products for MPCC‐Cu reaches ≈93.6%, whereas the FE_C2+_ for the Cu_2_O/Cu catalyst is less than 50%. Similarly, at −1.34 V, the FE_C2H4_ peaks at 83.3% for MPCC‐Cu, while that of the Cu_2_O/Cu catalyst is only 49.2%. Notably, FE_C2H4_ still remains ≈80% at ‐1.54 V. We speculate that this phenomenon may be attributed to the hygroscopic effect and micropores structure of the super‐hygroscopic porous nanosheets. Simultaneously, the competing reaction of H_2_ generation was well suppressed. This is attributed to electroneutrality, where OH^−^ balance the presence of K^+^. As the concentration of K^+^ increases (Figure S17, Supporting Information), the concentration of OH^−^ also rises accordingly, resulting in local enrichment of K^+^ and OH^−^. Consequently, this inhibits competitive hydrogen evolution reactions.^[^ ^41^, ^42^ ^]^ Figure 4d illustrates the comparative analysis of current densities for both catalysts at various potentials. Obviously, MPCC‐Cu consistently exhibited higher current densities compared to the Cu_2_O/Cu catalyst. For example, at ‐1.34 V, the current density reaches −123 mA cm^−2^ for MPCC‐Cu, while the Cu_2_O/Cu catalyst is −83 mA cm^−2^. This significant disparity further demonstrates the superior electrocatalytic performance of MPCC‐Cu over Cu_2_O/Cu catalyst. To elucidate the role of super‐hygroscopicity and the microporous structure, we conducted online GC measurements on CC‐Cu (Figure S18, Supporting Information). The results revealed that CC‐Cu exhibited significantly lower selectivity for ethylene compared to MPCC‐Cu (FE_C2H4_ = 42.5% vs. 83.3% for MPCC‐Cu at −1.34 V). This finding underscores that the super‐hygroscopicity and porous architecture of MPCC‐Cu play a crucial role in modulating ethylene selectivity.

The CO_2_RR performance of MPCC‐Cu was also evaluated in the MEA system (Figure S19, Supporting Information). MPCC‐Cu exhibits a volcanic distribution of FE_C2H4_, displaying a wide range of current densities in the MEA system. Notably, the maximum FE_C2H4_ reaches an impressive 96% at −200 mA cm^−2^ (Figure 4e; Figure S20, Supporting Information), surpassing all previously studied CO_2_RR electrocatalysts (Figure 4g; Table S4, Supporting Information). Furthermore, a CO_2_ single‐pass carbon efficiency (SPCE) of over 70% is achieved at −400 mA cm^−2^ (Figure 4f). The large fluctuations in the voltage‐time (V‐t) curves with increasing time at the same current density during the MEA system test may be due to the deposition of carbonates in the MPL of carbon paper, which limits the transmission of CO_2_ (Figure S21, Supporting Information). This can be easily solved by cleaning the GDE.^[^ ^43^ ^]^ Stability testing was conducted using constant current electrolysis technology in the MEA system, wherein CO_2_ was injected into a 0.5 M KHCO_3_ solution before entering the cathode of the MEA system, and 1 M KOH was utilized for the anode electrolyte. This test is evaluated at a current density of −200 mA cm^−2^. It was found that stability was maintained for 170 h throughout the test period. FE for ethylene remained stable, averaging ≈95% over the entire duration and consistently exceeding 90% for 168 h (Figure 4h; Table S1, Supporting Information). Subsequent to the long‐term performance testing, we further carried out a battery of catalyst characterizations. The results indicate that the catalyst had similar characteristics to MPCC‐Cu (Figures S22–S24, Supporting Information). The experimental results show that MPCC‐Cu exhibits excellent performance in ethylene synthesis, and its FE of ethylene is higher than most Cu catalysts reported in the literature. These findings provide beneficial insights for the design and development of efficient ethylene synthesis catalysts.

### 2.4 Theoretical Calculation, Exploration of Intermediates and Techno‐Economic Analysis

**Figure 5:**
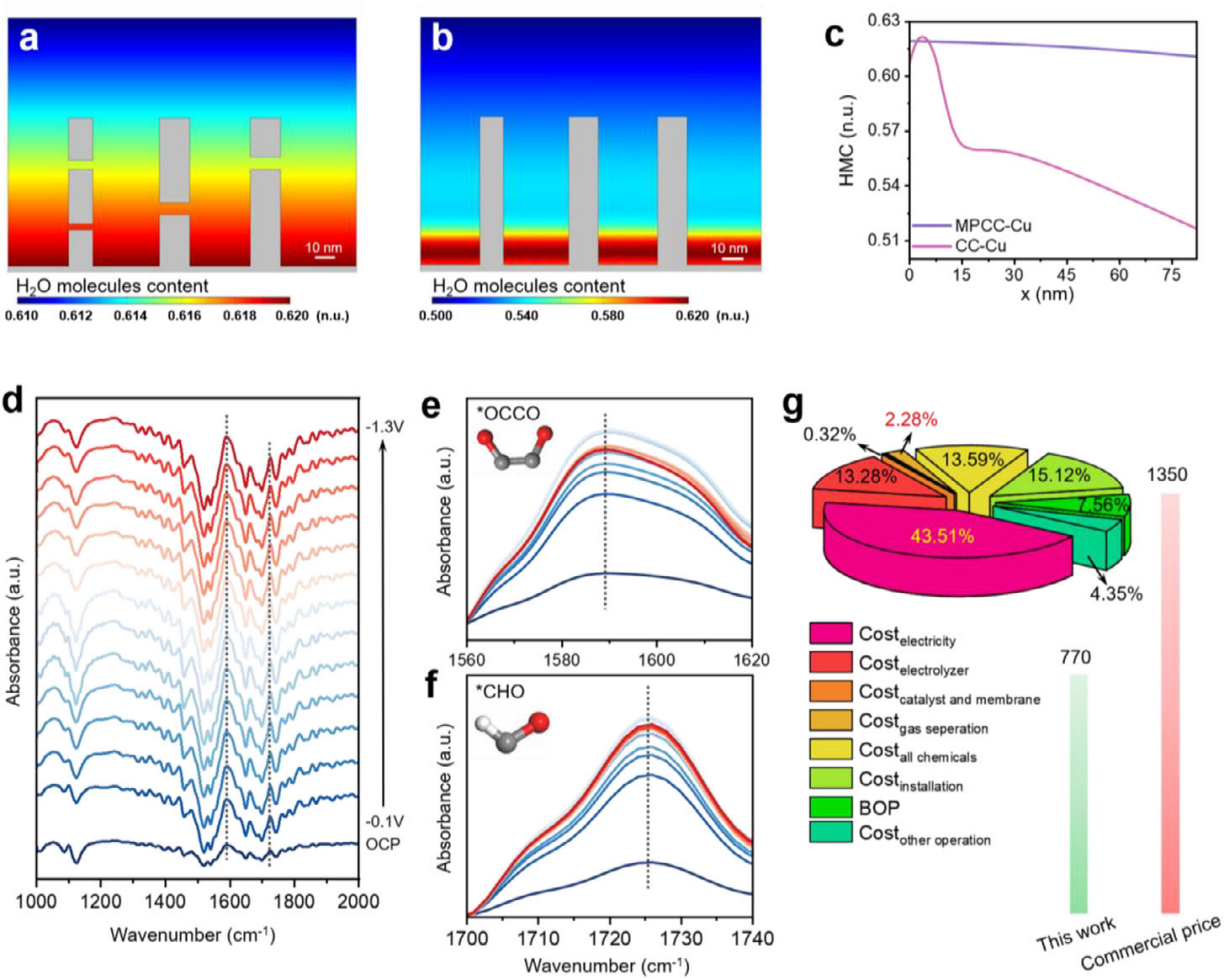
Theoretical calculations and in situ ATR‐FTIR testing. a,b) 2D distribution of HMC for MPCC‐Cu (a) and CC‐Cu (b). c) The 1D curve corresponds to (a) and (b). n.u., normalized unit. d) In situ ATR‐FTIR spectrum of MPCC‐Cu from 1000 to 2000 cm^−1^. e,f) In situ ATR‐FTIR spectra of MPCC‐Cu after amplification from 1560 to 1620 cm^−1^ (e) and 1700 to 1740 cm^−1^ (f). The test was conducted after CO_2_ was introduced into the 0.1 M KHCO_3_ solution for 20 min. g) The percentage of costs for electricity, electrolyzer, catalyst and membrane, gas separation, input chemicals, installation, balance of plant, and other operations to the total cost.

To further explore the effect of catalysts on H_2_O molecule content (HMC) distribution, we performed numerical simulations. Based on prior SEM characterization revealing micrometer‐scale lengths of the porous nanosheets (Figure 1c,d), we strategically modeled a reduced length of ≈80 nm to optimize computational efficiency while maintaining physical relevance. The inter‐sheet spacing was initialized at 35 nm with the outermost nanosheets remaining parallel, consistent with experimental observations. The funnel‐like architecture inherently generates progressively decreasing interlayer spacing toward the interior, which would induce confinement‐enhanced capillary condensation effects through geometrically modulated van der Waals interactions.^[^ ^26^ ^]^ This multiscale modeling approach faithfully represents the experimentally observed system while enabling precise interrogation of nanoconfined fluid dynamics, thereby providing a robust foundation for investigating structure‐dependent capillary phenomena in layered nanomaterials. **Figure** **5**a,b show the distribution of HMC for two different catalysts, respectively. Dark red (blue) indicates high (low) HMC. Compared with CC‐Cu, due to the micropores present on the surface of MPCC‐Cu, the HMC distribution of MPCC‐Cu is highly uniform on the nanosheets (Figure 5c). Therefore, we speculate that a more uniform distribution of HMC is more conducive to CO_2_RR. It provides sufficient proton sources and carbon sources for CO_2_RR.

To gain insight into the intermediate adsorption and C‐C coupling on MPCC‐Cu, we performed the in situ attenuated total reflection Fourier‐transform infrared spectroscopy (ATR‐FTIR) tests by multiple chronoamperometric scans under the real‐time CO_2_RR conditions. And it was based on a self‐made triple‐electrode electrolytic cell (Figure S25, Supporting Information). The spectra were collected from −0.1 to −1.3 V in the 0.5 M KHCO_3_ electrolyte saturated with CO_2_. The absorption peaks at 1589 cm^−1^ were observed with the increase in applied potential, corresponding to the key intermediate ^*^OCCO species in the formation of C_2_H_4_ (Figure 5d,e).^[^ ^44^ ^]^ The peak intensity initially rises before declining. It indicates that the high coverage of ^*^OCCO intermediate adsorbed on the catalyst surface is conducive to the generation of C_2_H_4_.^[^ ^45^ ^]^ However, the rapid depletion of ^*^OCCO in further reactions will lead to a decrease in the intensity of ^*^OCCO peaks.^[^ ^46^ ^]^ Simultaneously, the absorption peak at 1726 cm^−1^ is also observed, corresponding to ^*^CHO species,^[^ ^47^ ^]^ a significant intermediate following the protonation of surface‐bound CO (Figure 5d,f). It can also be attributed to the presence of multiple adsorbed CO.^[^ ^48^ ^]^ Its trend is consistent with the evolution of ^*^OCCO species. It can be inferred that the catalytic sites on MPCC‐Cu enhance C‐C coupling.

To comprehensively evaluate the economic feasibility of MPCC‐Cu for converting CO_2_‐to‐C_2_H_4_ during CO_2_RR. A detailed techno‐economic analysis (TEA) was conducted based on our experimental results (Table S3, Supporting Information). From our experimental results, it can be seen that when the current density is certain, the difference between FE_C2H4_ and the electricity cost both have a great impact on the total cost (Figure S26, Supporting Information). As shown in Figure 5g, the cost of gas separation is low, accounting for only 2.28% of the total cost. However, the cost of electricity accounts for 43.51% nearly half of the total cost. Therefore, we need to choose a lower price of electrical energy to achieve a higher economic value. This produces an economic value of ≈$580 per tonne of ethylene at current market prices when the electricity price is $0.01/kWh.

## 3 Conclusion

In summary, we propose the strategy to construct H_2_O and CO_2_ channels and achieve high selectivity and stability for ethylene. The synthesized MPCC‐Cu exhibits capillary condensation, which can condense H_2_O(g) into H_2_O(l), providing a pathway for proton transport and thus bolstering proton sources. Moreover, the microporous structures on MPCC‐Cu further provide channels for the diffusion transport of CO_2_ molecules and enrich carbon sources. As revealed by multiphysics simulations, XAS, and TOF‐SIMS results, the presence of H_2_O and micropores promotes the mass transport of protons and carbon sources, respectively. Thus, the selectivity of ethylene achieves ≈96% in the MEA system, while demonstrating continuous operation for over 170 h at −200 mA cm^−2^. The TEA suggests that the CO_2_‐to‐C_2_H_4_ system has a path to becoming profitable. This work provides a promising pathway to enhance the selectivity and stability of copper catalysts for ethylene production.

## Conflict of Interest

The authors declare no conflict of interest.

## Supporting information



Supporting Information

## Data Availability

The data that support the findings of this study are available on request from the corresponding author. The data are not publicly available due to privacy or ethical restrictions.
