# Supplementary material for: Accelerate Mass Transport of Proton and Carbon Sources by Super‐Hygroscopic and Porous Nanosheets for Continuous CO2‐To‐Ethylene Upgrade
Source: Adv Sci (Weinh). 2025 May 14;12(28):2502306. doi: 10.1002/advs.202502306 (PMC12302532; doi:10.1002/advs.202502306)
Supplement: Supplementary file 1 — Supporting Information [file ADVS-12-2502306-s001.docx]

Supplementary Information for

**Accelerate mass transport of proton and carbon sources by super-hygroscopic and porous nanosheets for continuous CO_2_-to-ethylene upgrade**

*Silong Dong, et al.*

**This file includes:**

1. Methods

2. Supplementary Figures S1-S26

3. Supplementary Tables S1-S4

4. References

1. Methods

**1.1. Synthetic procedures**

Preparation of CC-Cu and MPCC-Cu catalysts

CuSO_4_·5H_2_O was added to distilled deionized water (DDW) to prepare a 0.1 M solution. 2 M H_2_SO_4_ was added to make the solution pH=2. Ultrasonically treated the carbon paper (YLS-30T, Toray, Japan), and then added the prepared CuSO_4_ solution into the electrolytic cell. A three-electrode setup was used, consisting of treated carbon paper as the working electrode, a platinum electrode as the counter electrode, and a saturated calomel electrode as the reference electrode. Electrodeposition was performed using a potentiostat method at -0.72 V (vs. SCE) for 600 s. After the electrodeposition was completed, the electrode was taken out and rinsed with DDW and ethanol, and finally dried to obtain a Cu electrode. 3 M NaOH and 0.15 M (NH_4_)_2_S_2_O_8_ were mixed to obtain an etching solution. Soaked the Cu electrode in the etching solution for 4 hours, then took it out and washed it with DDW and ethanol. It was then dried in a vacuum oven at 60°C for 4 h to produce the CC-Cu catalyst. CC-Cu was reduced for 20 minutes using a three-electrode device to finally obtain MPCC-Cu.

Preparation of Cu_2_O/Cu catalyst

The Cu_2_O/Cu catalyst was synthesized through a facile thermal oxidation approach, wherein commercial copper powder was directly subjected to calcination in a muffle furnace or tube furnace at 200 °C for 2 hours with a controlled heating rate of 2.5°C min^-1^, followed by natural cooling to ambient temperature. The resulting catalyst was thoroughly ground to ensure homogeneity prior to electrode fabrication. For the preparation of catalytic electrodes, a precisely measured 5 mg aliquot of the catalyst was dispersed in 1 mL of a binary solvent system comprising isopropanol and deionized water (volume ratio of 7:3), followed by the addition of 80 μL Nafion solution (5 wt.%) as a binder. The mixture was subjected to vigorous stirring and subsequent ultrasonication for 1 hour to achieve a homogeneous ink. The resulting ink was then uniformly drop-cast onto carbon paper substrate and dried under ambient conditions to obtain the Cu_2_O/Cu catalytic electrode.

**1.2. Electrochemical measurement**

CO_2_RR was performed in a gas-tight H-cell and an MEA system. H-type cell had a Nafion 211 membrane which was sandwiched between the two PTFE sheets to divide the two compartments, preventing reoxidation of CO_2_RR products at the anode. Both catholyte and anolyte were 0.5 M KHCO_3_ solution, Same as previous work. CO_2_RR experiments were conducted in the cathode chamber filled with a CO_2_-saturated 0.5 M KHCO_3_ solution. Before testing, the KHCO_3_ electrolyte was purged with CO_2_ for 30 minutes until saturation was achieved. The converting formula was E (RHE) = E (SCE) + 0.235 V + 0.0591 × pH, in which the pH value was measured in 0.5 M CO_2_-saturated KHCO_3_ solution (pH = 7.2). The MEA system consisted of anode and cathode flow field plates with a geometric flow area of 5 cm^2^. The Nafion 117 membrane was used as a cation exchange membrane (CEM). The CEM was mounted between the cathode and anode for MEA system. Gaseous CO_2_ was fed to the cathode side through a humidification device with 0.5 M KHCO_3_ at a flow rate of 120 ccm, while 1.0 M KOH was fed to the anode side using a peristaltic pump at a rate of 10 mL min^-1^. Perform constant current electrochemical testing using the electrochemical workstation of CHI 660E. The current densities in this work were normalized based on the geometric surface area. The partial current densities were equal to the total current densities multiplied by the Faradaic efficiency. Three independent measurements were conducted, and the reported results are the averages. All measurements were performed under normal atmospheric conditions and at room temperature.

**1.3. Products analysis**

Liquid products in the catholyte were analyzed by the water suppression method on a nuclear magnetic resonance spectroscopy (400 MHz ^1^H liquid NMR spectrometer, Bruker Avance NEO). After testing, 1 ml of electrolyte-containing product was collected from the cathode chamber. It was mixed with an internal standard of dimethyl formamide (DMF, Sigma-Aldrich, >99.9%) and D_2_O (Sigma-Aldrich, 99.9%), and then sealed for NMR measurements. The CO_2_RR measurements were performed using an electrochemical workstation (Bio-logic vmp3，CHI660E). Gas products were quantitatively analyzed using an online gas chromatograph (GC, model GC-2014 ATFSPL 230C) equipped with a packed molecular sieve column. The online GC is equipped with a flame ionization detector (FID 1) for CO and CH_4_, a flame ionization detector (FID 2) for C_2_H_4_, C_2_H_6_, and C_2_H_2_, and a thermal conductivity detector (TCD) for H_2_.

The Faradaic efficiency (FE) can be calculated as follows:

$$\begin{aligned} \text{FE (}\text{\%}\text{) = }\frac{\text{znF}}{\text{Q}}\text{ × }100\%\#\left( \text{ AUTONUM \textbackslash* Arabic } \right) \end{aligned}$$

Where, z is the number of electrons exchanged in the reaction, n is the number of moles of product formed (obtained from the GC & NMR data using product calibration curves), F is the Faraday constant, Q is amount of charge passed.

**1.4. Characterization**

Scanning electron microscopy (SEM, ZEISS Sigma 500), atomic force microscopy (AFM, Bruker Dimension ICON), transmission electron microscopy (TEM), and high-resolution transmission electron microscopy (HRTEM) (FEI Talos F200x) were utilized to observe the morphology and crystal lattice of materials, respectively. The FEI Talos F200X TEM combines outstanding high-resolution S/TEM and TEM imaging with industry-leading dispersive X-ray spectroscopy (EDS) signal detection and 3D chemical characterization with compositional mapping. X-ray diffraction (XRD, Bruker/D8 ADVANCE X-ray diffractometer) and X-ray photoelectron spectrometer (XPS, Thermo Scientific K-Alpha XPS spectrometer) were performed to affirm the crystal structure and elemental composition. The elemental composition of various catalysts was studied using time-of-flight secondary ion mass spectroscopy (TOF-SIMS). To precisely analyze the dissemination of differently oriented crystal planes in the catalysts, the wide-angle X-ray scattering (WAXS) (Xenocs, Xeuss 3.0) tests were carried out. X-ray absorption spectroscopy (XAS) measurements of Cu K-edge were tested through SCI-GO (www.sci-go.com). To gain insight into the intermediate adsorption and C-C coupling on MPCC-Cu, the in-situ attenuated total reflection Fourier-transform infrared spectroscopy (ATR-FTIR) (NICOLET iS50 FT-IR) tests was performed.

**1.5. Multiphysics simulations**

As we reported, the content distribution of H_2_O molecules near the catalyst was simulated using a finite element solver based on COMSOL Multiphysics. The current module calculates the electric field as the opposite gradient of the potential V: E = - ∇ V. An electrolyte conductivity of 10 S/m was used, and all simulations utilized extremely fine triangular meshes.

**1.6. Techno-economic analysis**

To determine the economic capacity of using renewable electricity to produce ethylene through CO_2_RR, we conducted a Techno-economic Analysis (TEA) based on the revised model reported by previous studies.^[1]^ All parameters are listed in Supplementary Table S2.

In the model, we consider electricity, electrolyzer, catalyst and membrane, gas separation, input chemicals, installation, balance of plant and other operating costs. Due to differences in different regions, delivery costs are not considered here.

We investigated the cost of producing 100 tonnes of ethylene per day over a 20-year factory life with a production capacity factor of 0.9. We estimated the actual potential of a potential industrial electrolytic cell using the 2.8 V MEA full-cell voltage and a total current density of 200 mA/cm ² obtained from our stability experiment.

For the formation of ethylene from CO_2_, the chemical equation is:

$${2CO}_{2}+{8H}_{2}O\to C_{2}H_{4}+12OH^{-}-12e^{-}$$

$$4OH^{-}\to{O_{2}+2H}_{2}O+4e^{-}$$

$$2CO_{2}+2H_{2}O\to C_{2}H_{4}+{3O}_{2}$$

Therefore, producing 1 mol of ethylene requires 2 mol of CO_2_ and 2 mol of H_2_O. However, the selectivity of CO_2_-to-ethylene is less than 100%, so more than 2 mol of CO_2_ is required.

**1)、The cost of electricity**

The total current required to produce 100 tonnes of ethylene per day is:

$$Total current \left( kA \right)=\frac{\frac{\mathrm{ethylene} production \left( \frac{g}{day} \right)}{M_{\mathrm{ethylene}}\left( \frac{g}{mol} \right)}\times n\times F}{{FE}_{\mathrm{ethylene}}}= \frac{\frac{100\times\frac{{10}^{6}g}{day}}{\frac{28g}{mol}\times\frac{86400s}{day}}\times12\times96485\frac{C}{mol}}{96\%}=49859 kA$$

We multiply by the experimentally-derived MEA full-cell voltage (2.8 V) to get the power consumed:

$$Power consumed \left( kW \right)= Total current (kA)\times V_{\mathrm{cell}} \left( V \right)=139604 kW$$

By multiplying this required energy by the electricity price (1 ¢/kWh)^[1d]^ and dividing by the daily ethylene output, we determine the cost per tonne of ethylene:

$${Cost}_{electricity}\left( \frac{\$}{tonne ethylene} \right)=\frac{Power consumed (kW)\times{24 hours\times price}_{electricity}}{ethylene production (\frac{tonne ethylene}{day})}=\frac{139604 kW\times24 hours\times\frac{0.01\$}{kWh}}{100\left( \frac{tonne ethylene}{day} \right)}\mathbf{=3}\boldsymbol{35.05}\frac{\boldsymbol{\$}}{\boldsymbol{tonne} \boldsymbol{ethylene}}$$

**2)、The cost of electrolyzer**

The reference electrolyzer cost is 300 $/kW,^[1a]^ and the reference current density of 200 mA/cm².^[1b, 1c]^ Thus, we obtain the total cost of the electrolyzer:

$${Cost}_{Total electrolyzer}\left( \$ \right)=Power Consumed \left( kW \right)\times{Reference Cost}_{\mathrm{electrolyzer}} \left( \$ \right)\times\frac{base current density\left( \frac{mA}{cm^{2}} \right)}{input current density\left( \frac{mA}{cm^{2}} \right)}= 139604 kW\times300 \frac{\$}{\mathrm{kW}}\times\frac{200\left( \frac{mA}{cm^{2}} \right)}{200\left( \frac{mA}{cm^{2}} \right)}=\$41881200$$

The above is a one-time total cost of the electrolyzer, which must be converted to a cost per tonne of ethylene. This technique of converting long-term investments into daily costs is applied for all capital costs and employs a capital recovery factor (CRF) based on the discount rate i (5 %) and the lifetime of the material (20 years). The cost of electrolytic cells per tonne of ethylene is:

$${Cost}_{electrolyzer} \left( \frac{\$}{tonne ethylene} \right)=\frac{{CRF}_{electrolyzer}\times{Cost}_{electrolyzer} \left( \$ \right)}{Capacity factor\times365\left( \frac{days}{year} \right)\times production\left( \frac{tonne ethylene}{day} \right)}$$

Among them, due to a production capacity coefficient of 0.9, the factory life is 20 years. We can obtain:

$${CRF}_{electrolyzer}=\frac{i{(1+i)}^{lifetime}}{{(1+i)}^{lifetime}-1}=\frac{0.05{(1.05)}^{20}}{{(1.05)}^{20}-1}=0.080243$$

Hence,

$${Cost}_{electrolyzer} \left( \frac{\$}{tonne ethylene} \right)=\frac{0.080243\times\$41881200}{0.9\times365\left( \frac{days}{year} \right)\times100\left( \frac{tonne ethylene}{day} \right)}= \mathbf{1}\boldsymbol{02.3}\frac{\boldsymbol{\$}}{\boldsymbol{tonne}\boldsymbol{ethylene}}$$

**3)、Catalyst and membrane costs**

For catalyst and membrane costs, we assume 5% of the electrolyzer cost. We use the same procedure as described above to find the cost per tonne of ethylene using a catalyst/membrane lifetime of 5 years:

$${CRF}_{catalyst and membrane}=\frac{i{(1+i)}^{lifetime}}{{(1+i)}^{lifetime}-1}=\frac{0.05{(1.05)}^{5}}{{(1.05)}^{20}-1}=0.038598$$

$${Cost}_{catalyst and membrane} \left( \frac{\$}{tonne ethylene} \right)=\frac{{CRF}_{C\&M}\times{Cost}_{Total electrolyzer} \left( \$ \right)\times5\%}{Capacity factor\times365\left( \frac{days}{year} \right)\times production\left( \frac{tonne ethylene}{day} \right)}=\frac{0.038598\times\$41881200\times5\%}{0.9\times365\left( \frac{days}{year} \right)\times100\left( \frac{tonne ethylene}{day} \right)}=\boldsymbol{2.46}\frac{\boldsymbol{\$}}{\boldsymbol{tonne} \boldsymbol{e}\boldsymbol{thylene}}$$

**4)、Cathode gas separation cost**

Gaseous side products (H_2_) are separated from the CO_2_ outlet stream to enable the recirculation of unreacted CO_2_. We calculate gaseous separation costs using estimates from a reference pressure-swing adsorption (PSA) system. The reference cost used in this model is $1989043, with a flow capacity of 1000 m^3^/h, a scaling factor of 0.7, and an energy consumption of 0.25 kWh/m^3^ (ref.^[1b, 1d, 2]^). The cathode output gas includes ethylene, CO_2_, and H_2_. At 200 mA/cm^2^, the FEs of ethylene and H_2_ are 96% and 3%, respectively.

$$Ethylene flow rate \left( \frac{m^{3}}{hour} \right)=\frac{ethylene production rate\times R\times T}{P}=\frac{\frac{100\times{10}^{6}}{28\times24} \frac{mol}{h}\times8.314 \frac{J}{mol K}\times298.15 K}{101 300 Pa}=3641.4 \frac{m^{3}}{hour}$$

$$Output {CO}_{2} flow rate \left( \frac{m^{3}}{hour} \right)=\frac{ethylene flow rate\times(1-Single pass rate)}{Single pass rate}=5462.1 \frac{m^{3}}{hour}$$

$$Output H_{2} Flow Rate \left( \frac{m^{3}}{hour} \right)=\frac{H_{2} production rate (\frac{mol}{s})\times R\times T}{P}=\frac{\frac{Total current\times{FE}_{H2}}{n\times F}\times R\times T}{P}=\frac{\frac{49859000\times3\%}{2 e^{-}\times96485} \left( \frac{mol}{s} \right)\times3600 s\times8.314 \frac{J}{mol K}\times298.15 K}{101300 Pa}=682.8\frac{m^{3}}{hour}$$

The sum of gaseous side product flow rates and the flow rate of unreacted CO_2_ is used to estimate the gaseous separation cost:

$$The total flow rate \left( \frac{m^{3}}{hour} \right)=\mathrm{Et}h\mathrm{ylene} flow rate\left( \frac{m^{3}}{hour} \right)+Output {CO}_{2} flow rate\left( \frac{m^{3}}{hour} \right)+Output H_{2} flow rate\left( \frac{m^{3}}{hour} \right)=9786.3 \frac{m^{3}}{hour}$$

$${Cost}_{PAS capital} \left( \frac{\$}{tonne ethylene} \right)=\frac{reference PSA capital cost\times{(\frac{total flow rate}{reference capacity})}^{scaling factor}\times CRF}{Capacity factor\times365\times target output production}=\frac{\$1989043\times{(\frac{9786.3\frac{m^{3}}{hour}}{1000 \frac{m^{3}}{hour}})}^{0.7}\times0.039}{0.9\times365\frac{day}{year}\times100 \frac{tonne ethylene}{day}}=\boldsymbol{11.66}\frac{\boldsymbol{\$}}{\boldsymbol{tonne} \boldsymbol{e}\boldsymbol{thylene}}$$

$${Cost}_{PSA operating} \left( \frac{\$}{tonne ethylene} \right)=\frac{total flow rate\times reference PSA Cost\times electricity price}{target output production}=9786.3\frac{m^{3}}{hour}\times24\frac{hour}{day}\times0.25\frac{kWh}{m^{3}} \times0.01 \frac{\$}{kWh}\div100 \frac{tonne ethylene}{day}=\mathbf{5}\boldsymbol{.87}\frac{\boldsymbol{\$}}{\boldsymbol{tonne} \boldsymbol{e}\boldsymbol{thylene}}$$

$${Cost}_{gas seperation} \left( \frac{\$}{tonne ethylene} \right)= {Cost}_{PAS capital} \left( \frac{\$}{tonne ethylene} \right)+{Cost}_{PSA operating} \left( \frac{\$}{tonne ethylene} \right)=\boldsymbol{1}\boldsymbol{7.53}\frac{\boldsymbol{\$}}{\boldsymbol{tonne} \boldsymbol{e}\boldsymbol{thylene}}$$

**5)、Input chemicals cost**

**Input CO_2_:**

Calculate the total CO_2_ input cost to produce 100 tonnes of ethylene at a market price of $30/tonne as:

$${Cost}_{\mathrm{intput}{CO}_{2}}\left( \frac{\$}{tonne ethylene} \right)= {price}_{CO2 market}\times{CO}_{2} required=={price}_{CO2 market}\times product output \left( \frac{tonne product}{day} \right)\times\frac{M_{{CO}_{2}}}{M_{product}}\times molar ratio \left( \frac{{CO}_{2}}{product} \right)=30\frac{\$}{tonne {CO}_{2}}\times314.3 \frac{tonne {CO}_{2}}{day}\times\frac{1}{100 tonne\frac{ethylene}{day}}=\mathbf{9}\boldsymbol{4.29}\frac{\boldsymbol{\$}}{\boldsymbol{tonne} \boldsymbol{e}\boldsymbol{thylene}}$$

**Input H_2_O:**

The total water input cost for producing 100 tonnes of ethylene is calculated at a market price of $5/tonne, as follows:

$${Cost}_{\mathrm{intput}H_{2}O}\left( \frac{\$}{tonne ethylene} \right)={price}_{H2O market}\times{H_{2}O}_{required}={price}_{H2O market}\times product output \left( \frac{tonne product}{day} \right)\times\frac{M_{H_{2}O}}{M_{product}}\times molar ratio \left( \frac{H_{2}O}{product} \right)=5\frac{\$}{tonne {CO}_{2}}\times128.57 \frac{tonne H_{2}O}{day}\times\frac{1}{100 tonne\frac{ethylene}{day}}= \mathbf{6}\boldsymbol{.43}\frac{\boldsymbol{\$}}{\boldsymbol{tonne} \boldsymbol{e}\boldsymbol{thylene}}$$

**Input KOH:**

For the cost of investing 1.0 M KOH, we have considered the consumption of H_2_O and KOH electrolyte costs. By using an electrolytic cell fixed at 100 L m^-3^, the total volume of electrolyte required can be calculated.

$${Volume}_{electrolyte} \left( L \right)= Surface {area}_{electrolyzer} \left( m^{2} \right)\times100 \left( \frac{L}{m^{2}} \right)=\frac{{Total}_{current} (A)}{Current density (\frac{mA}{{cm}^{2}})\times{(\frac{100 cm}{1m})}^{2}}\times100 \left( \frac{L}{m^{2}} \right)=\frac{{Total}_{current} (A)}{200 (\frac{mA}{{cm}^{2}})\times{(\frac{100 cm}{1m})}^{2}}\times100 \left( \frac{L}{m^{2}} \right)=2492950 L$$

$${Mass}_{KOH} \left( kg \right)={molarity}_{KOH} \left( \frac{mol}{L} \right)\times{Volume}_{electrolyte} \left( L \right)\times molecular weight \left( \frac{g}{mol} \right)=1\times2492950\times56 \left( g \right)=139605.2 kg$$

The price of KOH is $790 per tonne, so we can calculate the cost of the electrolyte.

$${Cost}_{KOH and water} \left( \$ \right)=790 \frac{\$}{tonne KOH}\times139.6 \frac{tonne KOH}{day}+2492950 L\times\frac{1}{1000}\frac{tonne}{L}\times5\frac{\$}{tonne}=\$122748.75$$

To obtain the cost per tonne of ethylene, we assume an electrolyte lifespan of one year to calculate the new CRF:

$${CRF}_{new}=\frac{i{(1+i)}^{lifetime}}{{(1+i)}^{lifetime}-1}=\frac{0.05{(1.05)}^{1}}{{(1.05)}^{1}-1}=1.05$$

Therefore, the electrolyte cost per tonne of ethylene is:

$${Cost}_{KOH and water} \left( \frac{\$}{tonne ethylene} \right)=\frac{{CRF}_{new}\times{Cost}_{KOH and water} \left( \$ \right)}{Capacity factor\times365 \left( \frac{days}{year} \right)\times production \left( \frac{tonne ethylene}{day} \right)}=\frac{1.05\times\$122748.75}{0.9\times365 \left( \frac{days}{year} \right)\times100 \left( \frac{tonne ethylene}{day} \right)}=\boldsymbol{3}\boldsymbol{.92}\frac{\boldsymbol{\$}}{\boldsymbol{tonne} \boldsymbol{e}\boldsymbol{thylene}}$$

**Total cost of all chemicals input:**

$${Cost}_{all chemicals} \left( \frac{\$}{tonne ethylene} \right)= {Cost}_{{input CO}_{2}}+{Cost}_{KOH and water}{+ Cost}_{input H_{2}O}=94.29+3.92+6.43=\mathbf{1}\boldsymbol{04.64}\frac{\boldsymbol{\$}}{\boldsymbol{tonne} \boldsymbol{e}\boldsymbol{thylene}}$$

**6)、The total capital costs**

All capital costs are adjusted to estimate the price of peripheral equipment surrounding the electrolyzer and separation units. To determine our total capital costs, we aggregate the expenses of the electrolyzer, catalyst and membrane, and cathode separation capital.

$${Cost}_{Total Capital} \left( \frac{\$}{tonne ethylene} \right)={Cost}_{electrolyzer}+{Cost}_{catalyst and membrane}+{Cost}_{PSA capital}=102.3+2.46+11.66=\boldsymbol{116.42}\frac{\boldsymbol{\$}}{\boldsymbol{tonne} \boldsymbol{e}\boldsymbol{thylene}}$$

**7)、The cost of installation**

We assume that the Lang factor is 1 to calculate the equipment installation cost based on the total capital cost.

$${Cost}_{installation} \left( \frac{\$}{tonne ethylene} \right)=Lang Factor\times{Cost}_{Total Capital}=1\times116.42\frac{\$}{tonne ethylene}\mathbf{=1}\boldsymbol{16.42}\frac{\boldsymbol{\$}}{\boldsymbol{tonne} \boldsymbol{e}\boldsymbol{thylene}}$$

**8)、Balance of plant (BOP)**

Assuming the balance of the factory is 50% of the total cost of capital.

$$BOP \left( \frac{\$}{tonne ethylene} \right)=BOP Factor\times{Cost}_{Total Capital}=0.5\times116.42\frac{\$}{tonne ethylene}=\mathbf{5}\boldsymbol{8.21}\frac{\boldsymbol{\$}}{\boldsymbol{tonne} \boldsymbol{e}\boldsymbol{thylene}}$$

**9)、Other operating costs**

To consider the additional operating costs associated with operating the factory (such as labor and maintenance), we have added an additional cost equal to 10% of the electricity cost:

$${Costs}_{other operation}\left( \frac{\$}{tonne ethylene} \right)={Cost}_{electricity}\left( \frac{\$}{tonne ethylene} \right)\times0.1=\mathbf{3}\boldsymbol{3.51}\frac{\boldsymbol{\$}}{\boldsymbol{tonne} \boldsymbol{e}\boldsymbol{thylene}}$$

**The overall expense for producing one tonne of ethylene:**By adding up all the above costs, we can obtain the cost required to produce one tonne of ethylene.

$${Cost}_{ethylene} \left( \frac{\$}{tonne ethylene} \right)={Cost}_{electricity}+{Cost}_{electrolyzer}{+Cost}_{catalyst and membrane}+{Cost}_{gas seperation}+{Cost}_{all chemicals}+{Cost}_{installation}+BOP+{Costs}_{other operation}=335.05+102.3+2.46+17.53+104.64+116.42+58.21+33.51=\mathbf{7}\boldsymbol{70.12}\frac{\boldsymbol{\$}}{\boldsymbol{tonne} \boldsymbol{e}\boldsymbol{thylene}}$$

2. Supplementary Figures


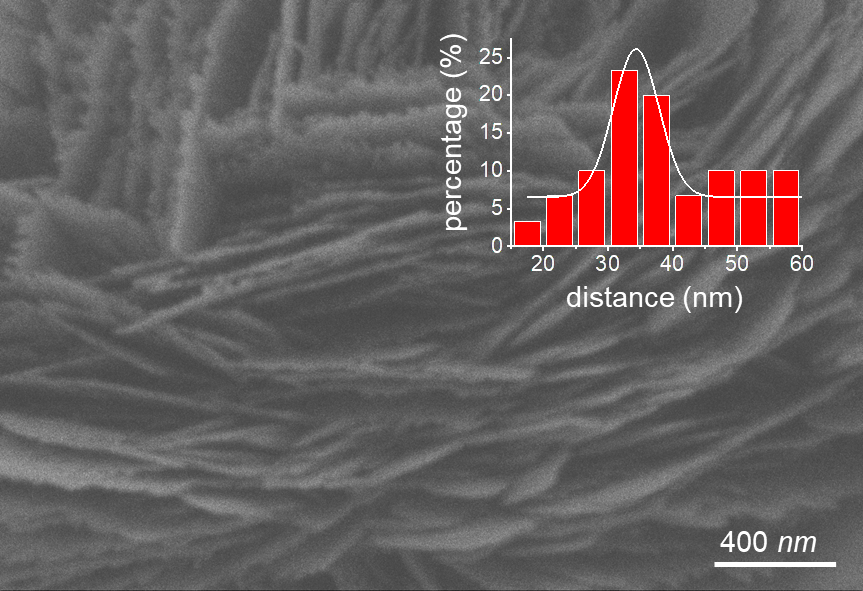


**Figure S1.** The illustration shows the distance distribution of adjacent nanosheets in this image.


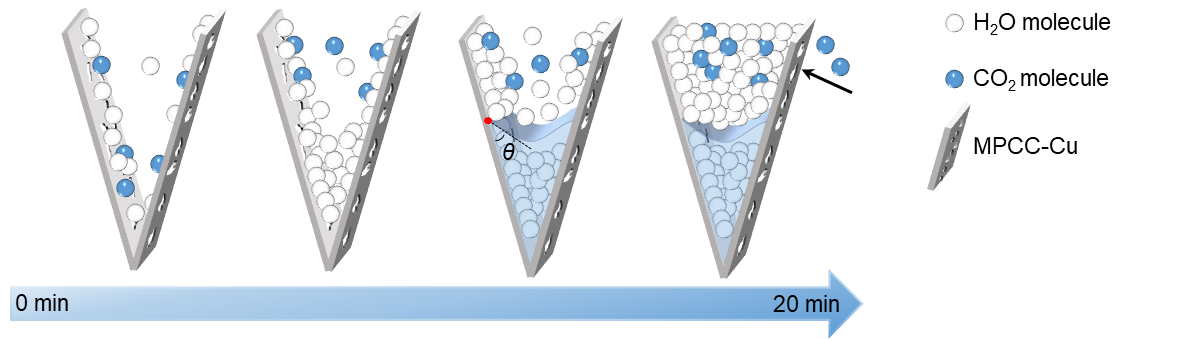


**Figure S2.** Schematic diagram of the formation process of triple-phase interface from 0 to 20 minutes with humid CO_2_ gas contacts MPCC-Cu.

$ln\frac{P}{P^{0}}=\frac{2\gamma M}{\rho RT}\cdot\frac{1}{r}$ (1)

$$d=2rcos \theta$$

where P/P^0^ is the relative pressure, γ represents the surface tension, M denotes the molar mass of CO_2_, ρ is the density of liquid CO_2_, R denotes the gas constant; T and r respectively stands for the absolute temperature and radius of curvature of the meniscus.^[3]^. When measuring the distance between two adjacent nanosheets. Suppose they are parallel and the distance is *d*. Then we can get$d=2rcos \theta$, where *r* is the meniscus curvature radius and *θ* is the water and catalyst contact angle of the surface (0°<*θ*<90°). *d* is a constant value, so *r* is proportional to *θ*.


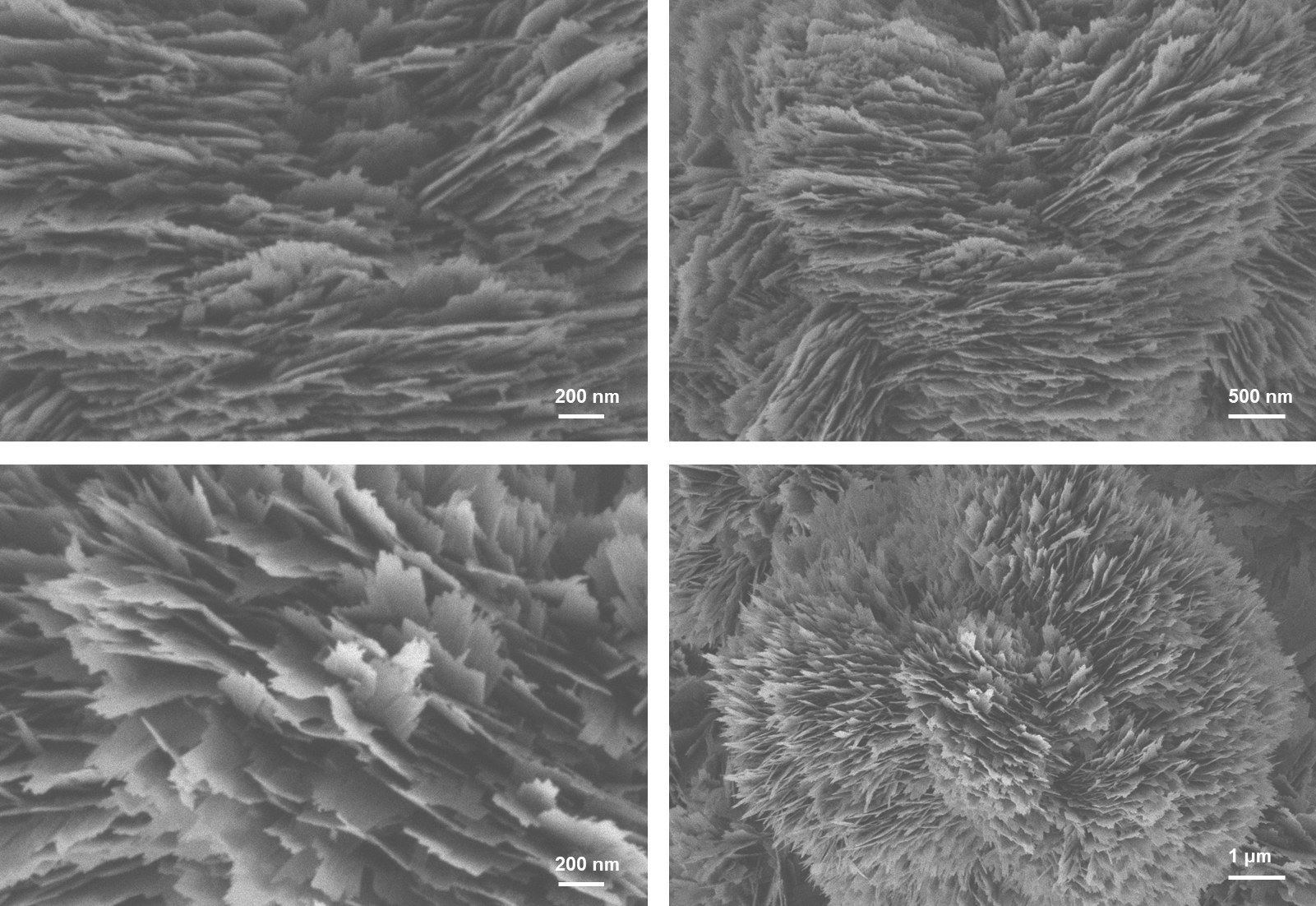


**Figure S3.** SEM images of CC-Cu.


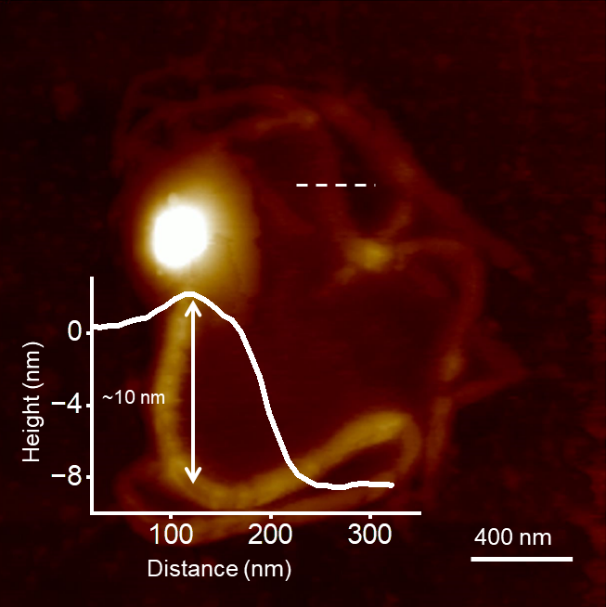


**Figure S4.** AFM image of CC-Cu.

We have tried several times to take AFM photos to solve the problem of presenting clear AFM images of microporous copper nanosheets at lower magnifications, showing their overall nanosheet morphology. However, since the catalyst was grown directly on carbon paper, during the AFM sampling process, we tried to directly scrape it off with a blade for ultrasonic treatment and ultrasonic treatment in anhydrous ethanol to obtain the nanosheets we wanted. Unfortunately, the nanosheets may be poorly hardened and broken during the ultrasonic treatment, so it is difficult to capture the complete nanosheets with micropores.


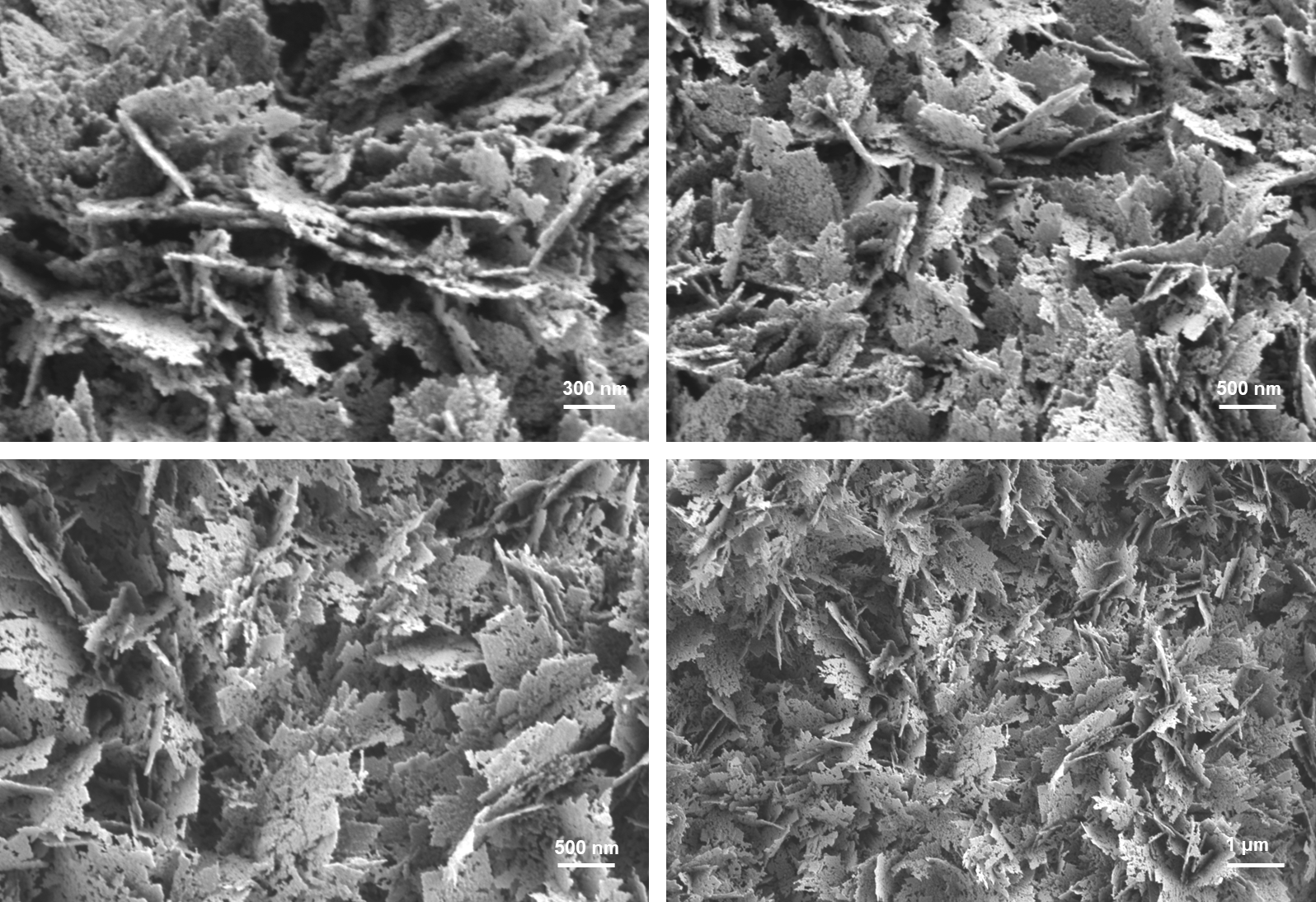


**Figure S5.** SEM images of MPCC-Cu.


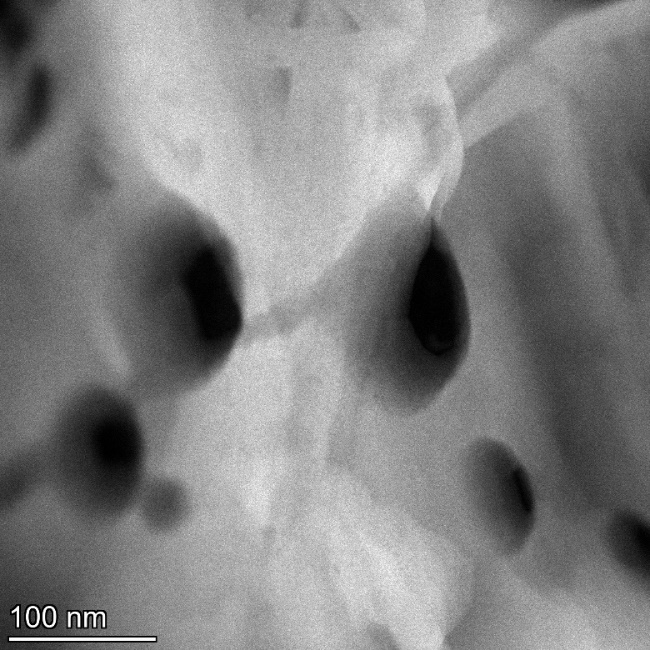


**Figure S6.** HRTEM images of MPCC-Cu.


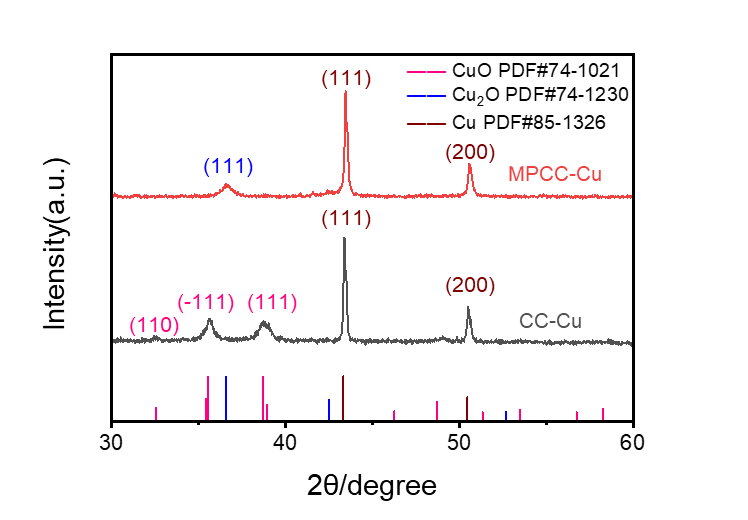


**Figure S7.** XRD analysis of CC-Cu and MPCC-Cu.


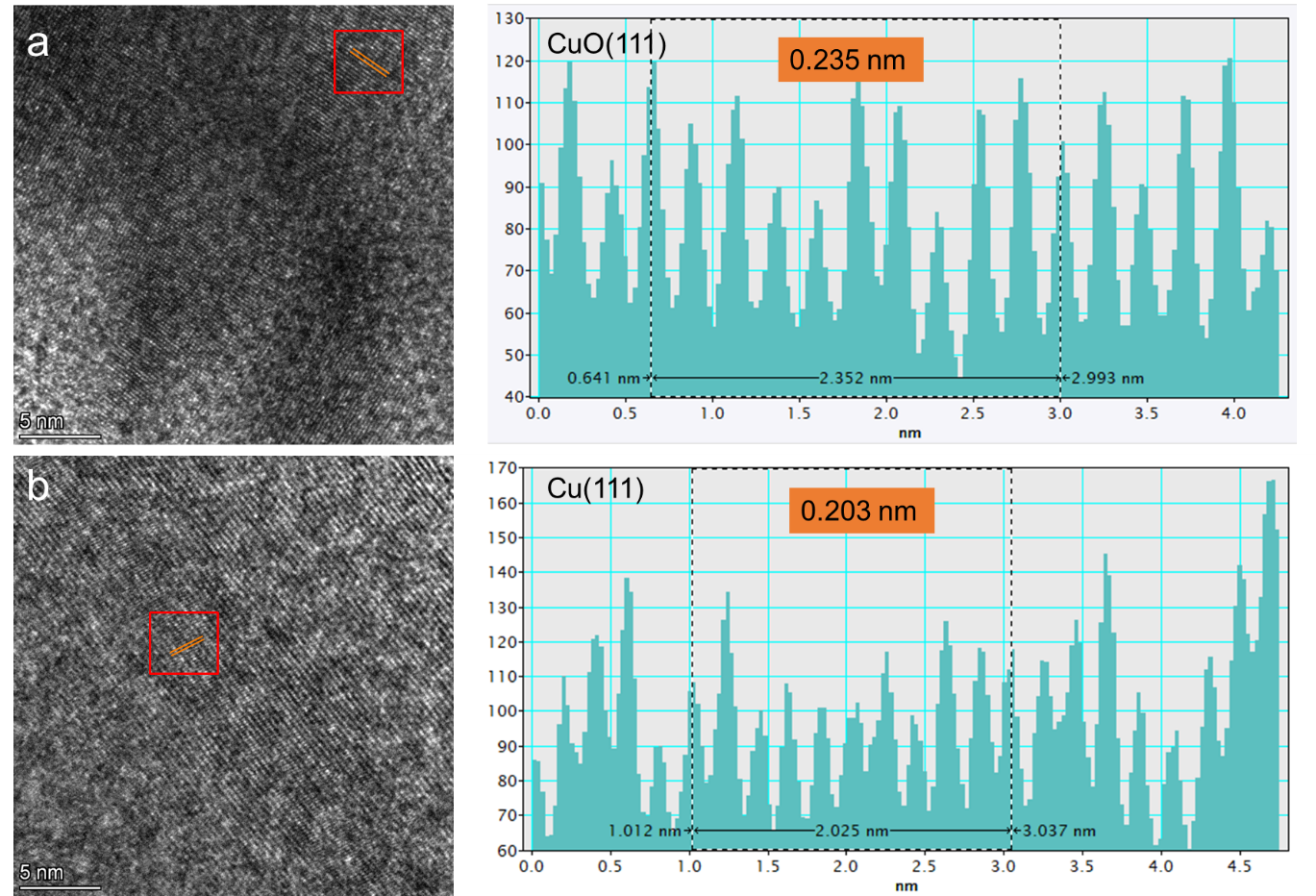


**Figure S8.** (a,b) HRTEM image of CC-Cu and the selected interplanar distance measurements.


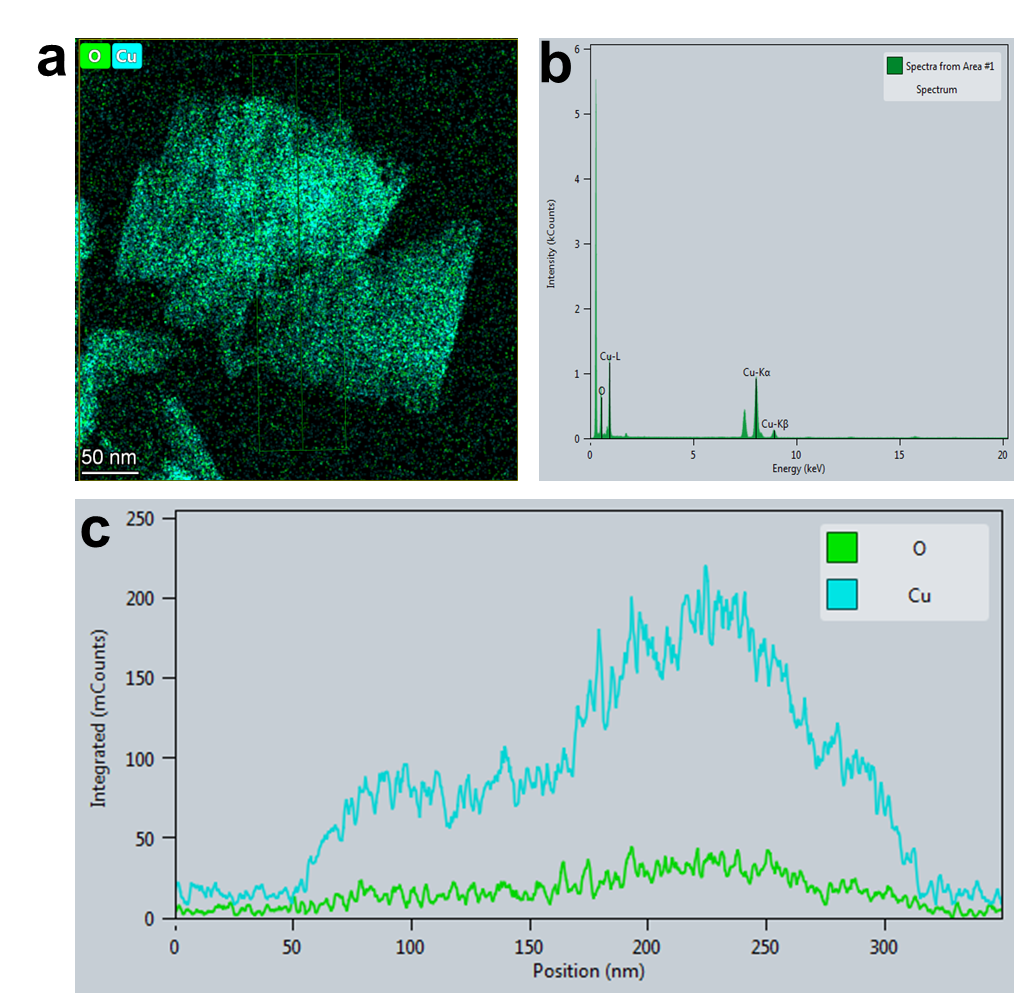


**Figure S9.** (a) HAADF-STEM image of the CC-Cu. (b) EDS spectrum and (c) EDS line scan corresponding to the line shown in (a).


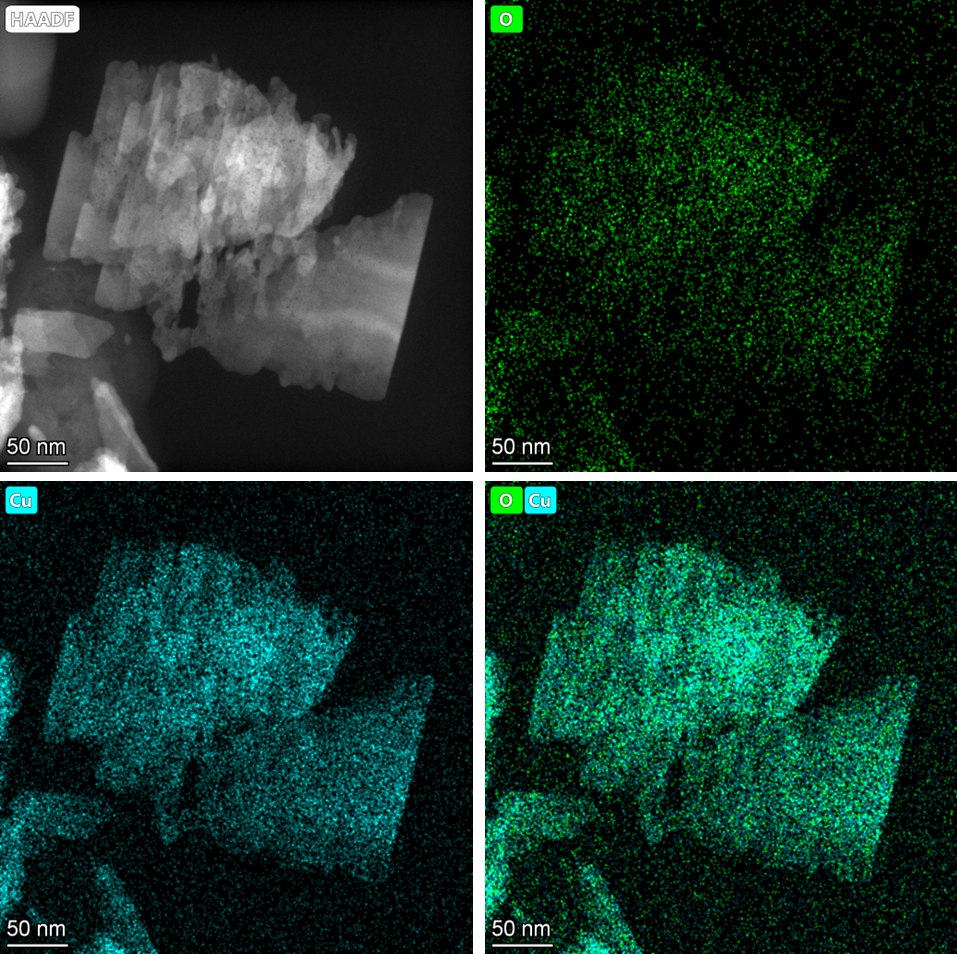


**Figure S10.** HAADF-STEM image and EDS element mapping of CC-Cu.


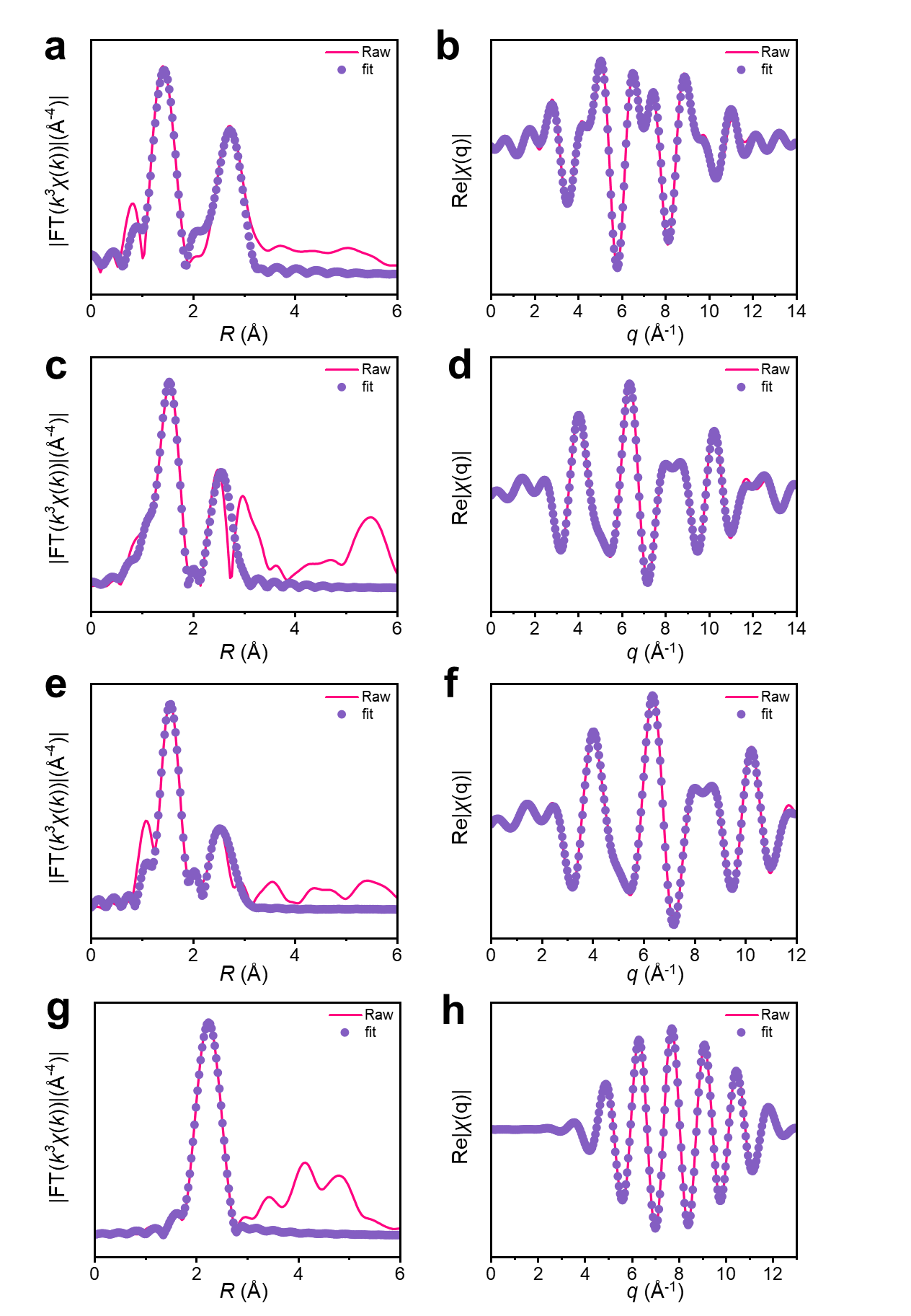


**Figure S11.** Fourier transform of Cu K-edge EXAFS oscillations in R space and K space the corresponding results of curve-fitting. (a,b) Cu_2_O. (c,d) CuO. (e,f) CC-Cu. (g,h) Cu foil.


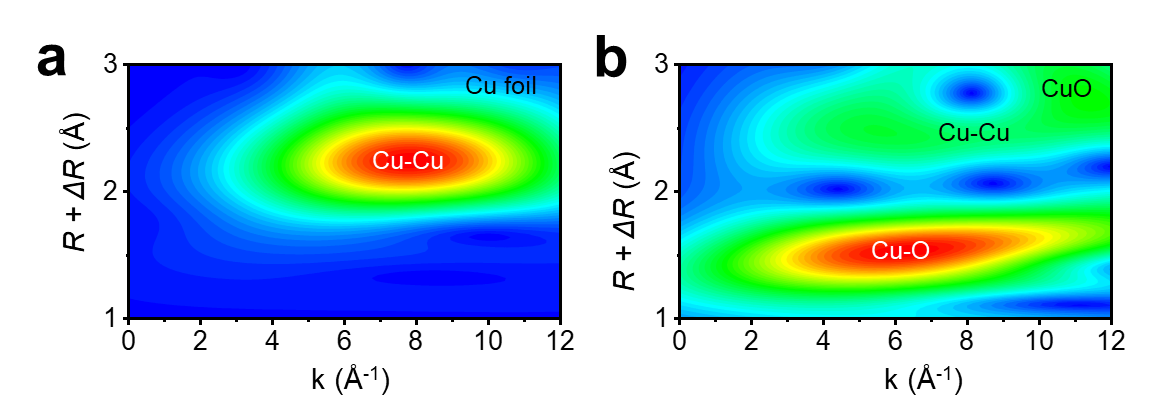


**Figure S12.** Wavelet-transformed k^3^-weighted EXAFS spectra of Cu foil (a) and CuO (b).


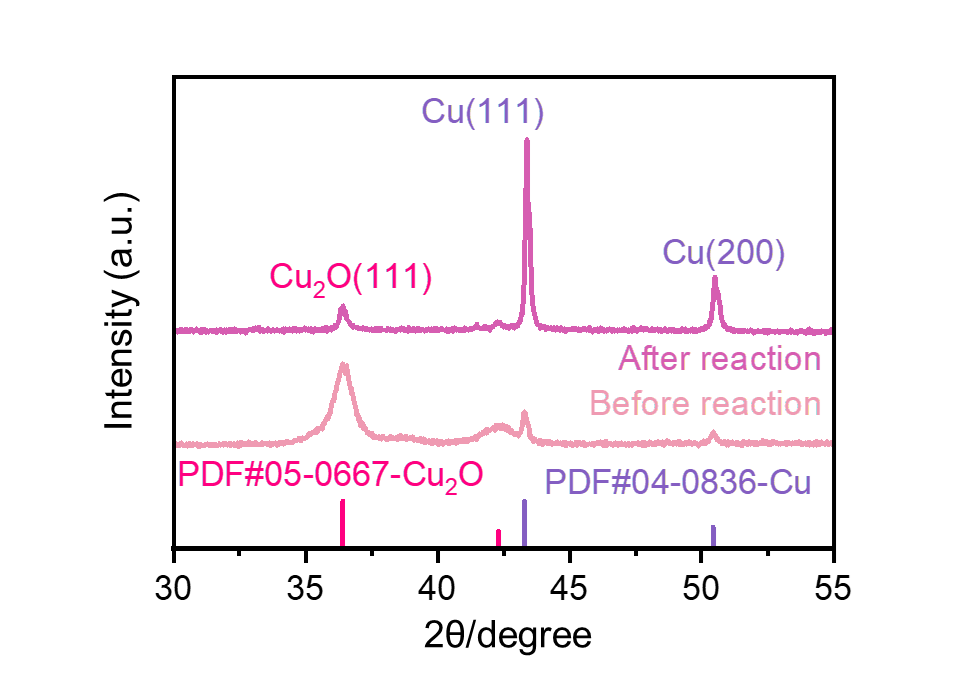


**Figure S13.** XRD analysis of Cu_2_O/Cu catalyst before and after reaction.


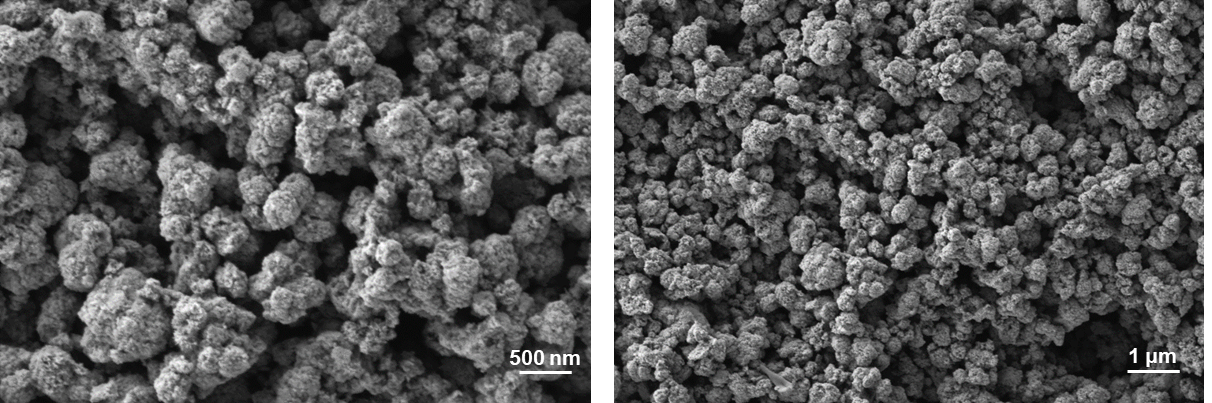


**Figure S14.** SEM images of Cu_2_O/Cu catalyst before reaction.


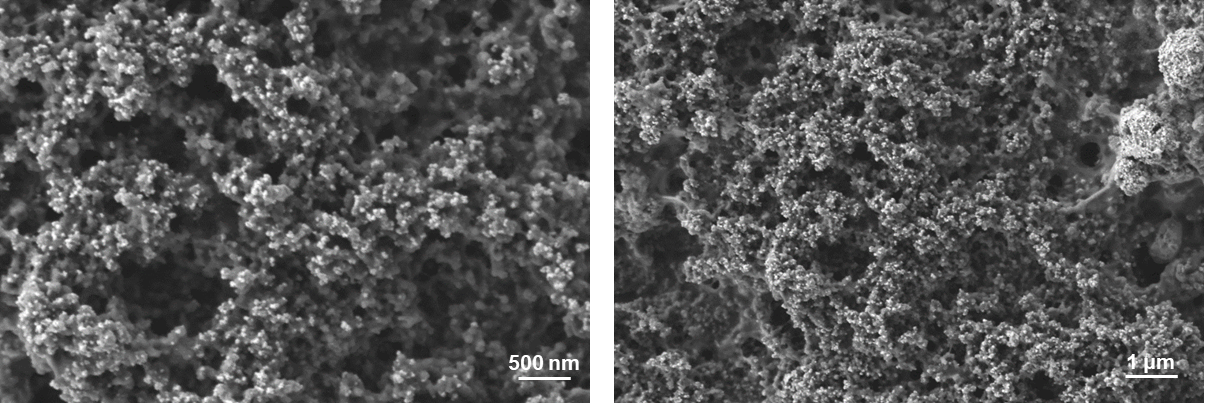


**Figure S15.** SEM images of Cu_2_O/Cu catalyst after reaction.


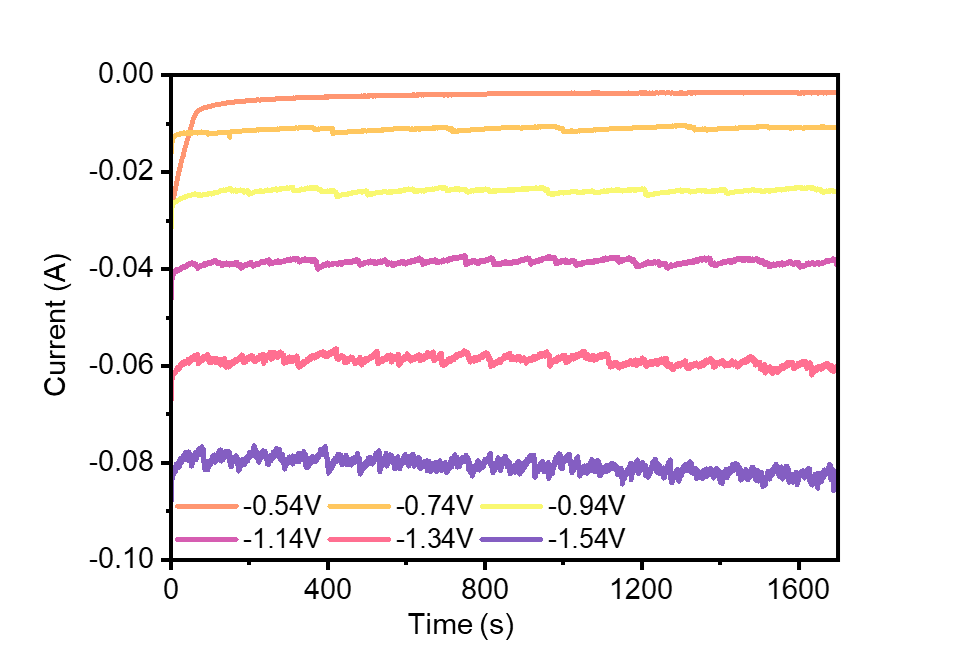


**Figure S16.** I-t curves of MPCC-Cu under different voltages during H-cell testing.


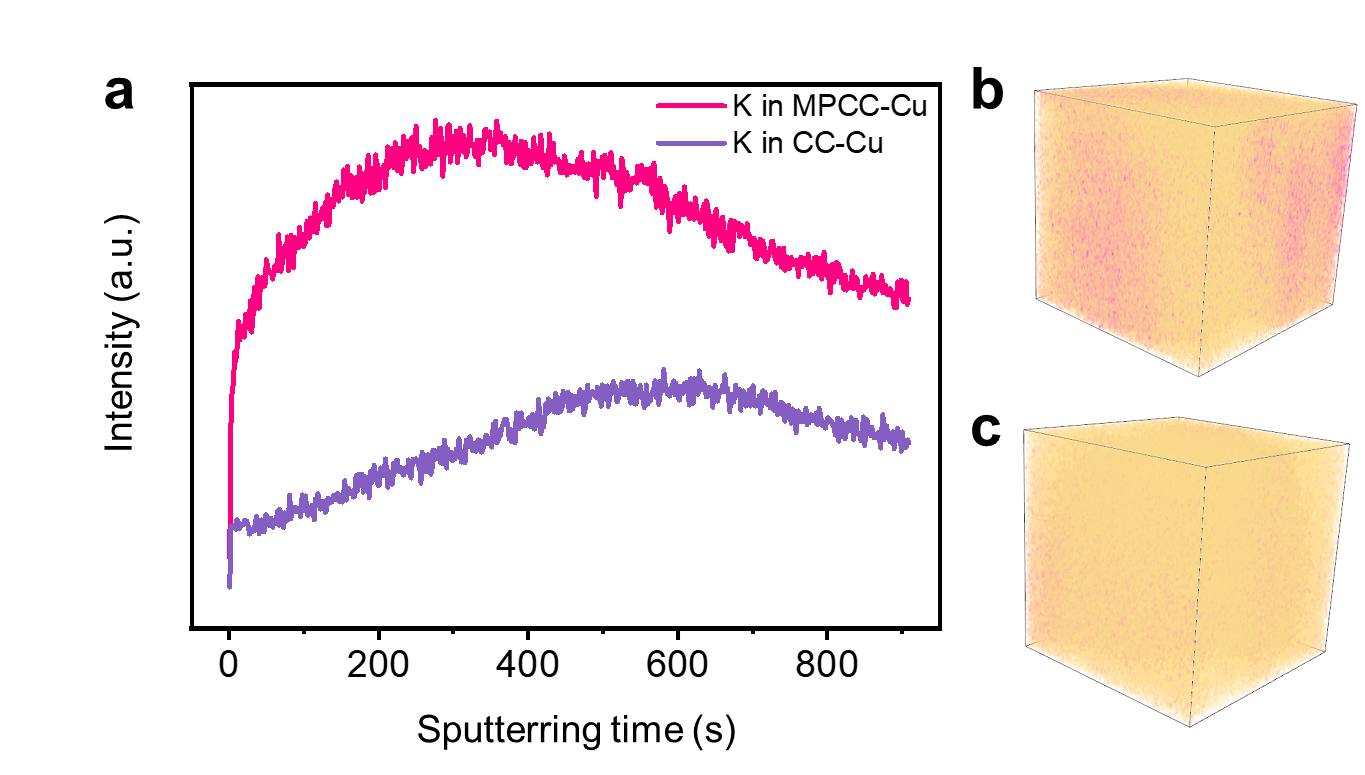


**Figure S17.** (a) TOF-SIMS depth profiles of K element for CC-Cu and MPCC-Cu. (b-c) TOF-SIMS 3D reconstruction of K element for MPCC-Cu (b) and CC-Cu (c).


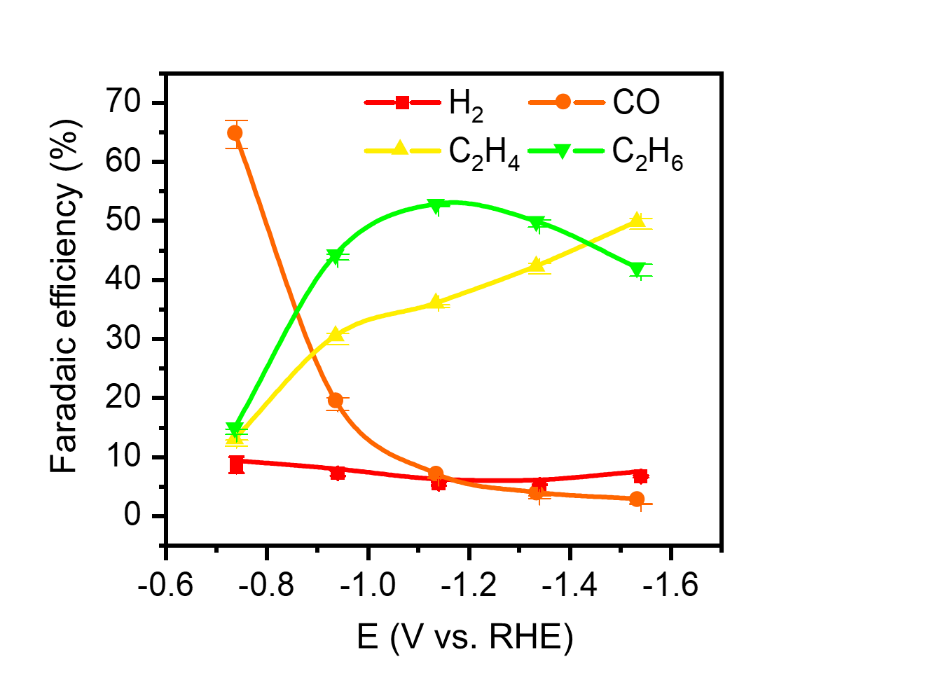


**Figure S18.** FE of various products of CC-Cu with potentials ranging from -0.64 to -1.54 V in the H-cell.


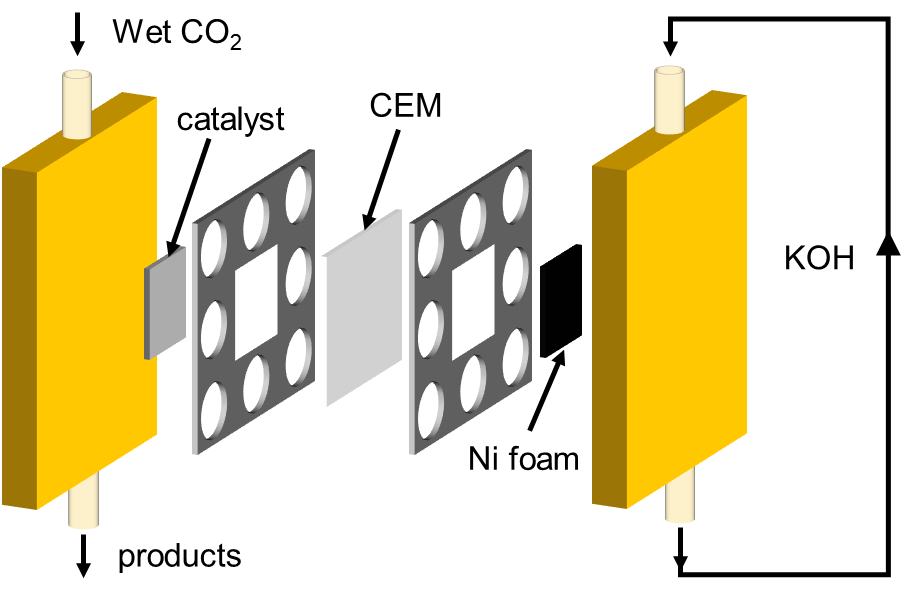


**Figure S19.** Schematic illustration of the MEA system (CEM: cation exchange membrane).

In the membrane electrode assembly (MEA), Nafion 117 served as the cation exchange membrane to regulate ion transport. Its sulfonic acid groups selectively facilitate H^+^ migration from anode to cathode, maintaining charge balance while blocking OH^-^ crossover. This proton conductive behavior preserves the alkaline microenvironment on the cathode side by accumulating OH^-^ ions, with compensatory K^+^ transport through the membrane preventing excessive negative charge buildup. Critically, the membrane physically separates H^+^ and OH^-^ to avoid water formation, thereby stabilizing the CO_2_ reduction reaction interface.^[4]^


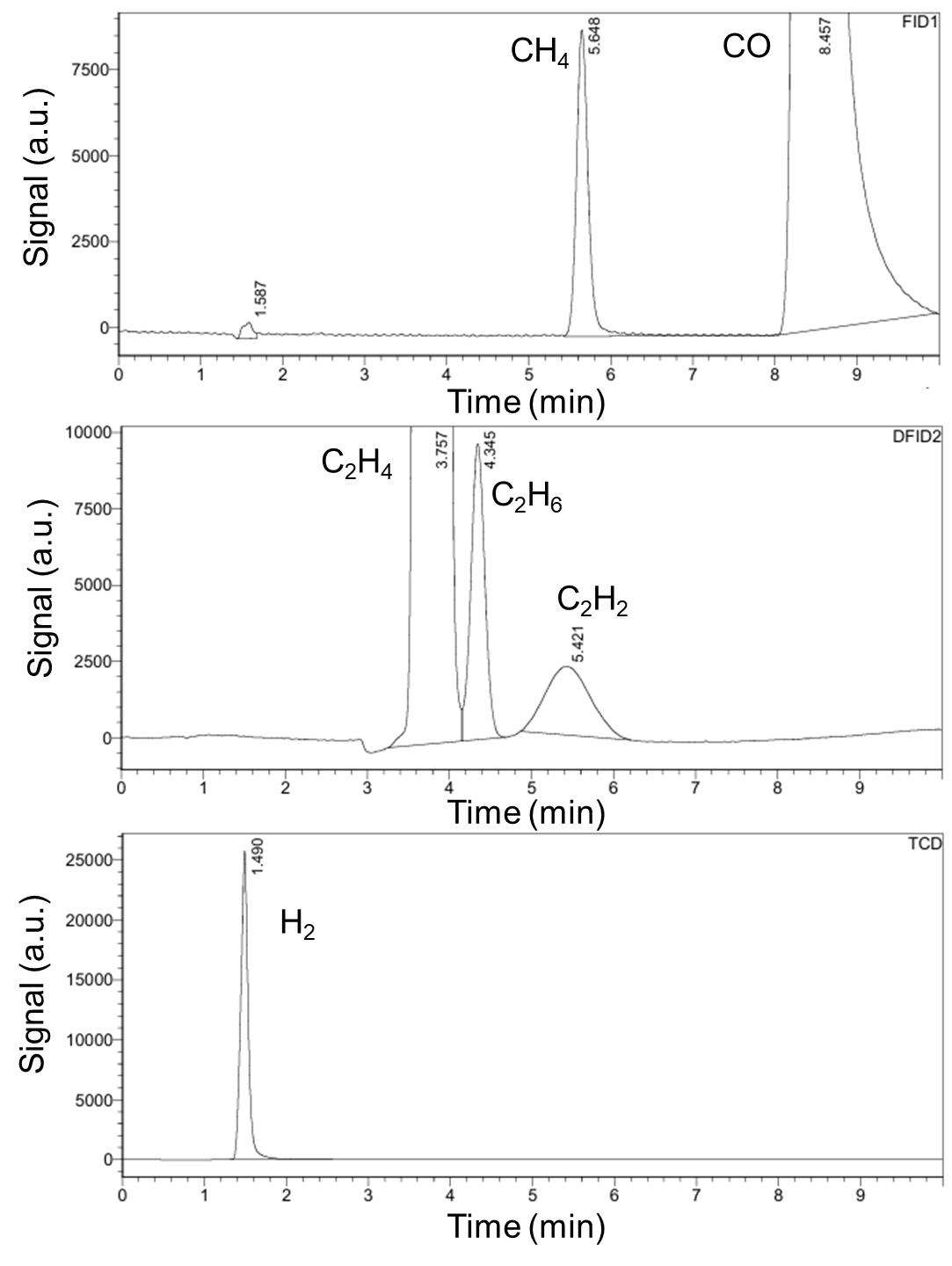


**Figure S20.** Chromatographic profiles of electrocatalytic performance tests under CO_2_ at -200 mA cm^-2^ for MPCC-Cu.


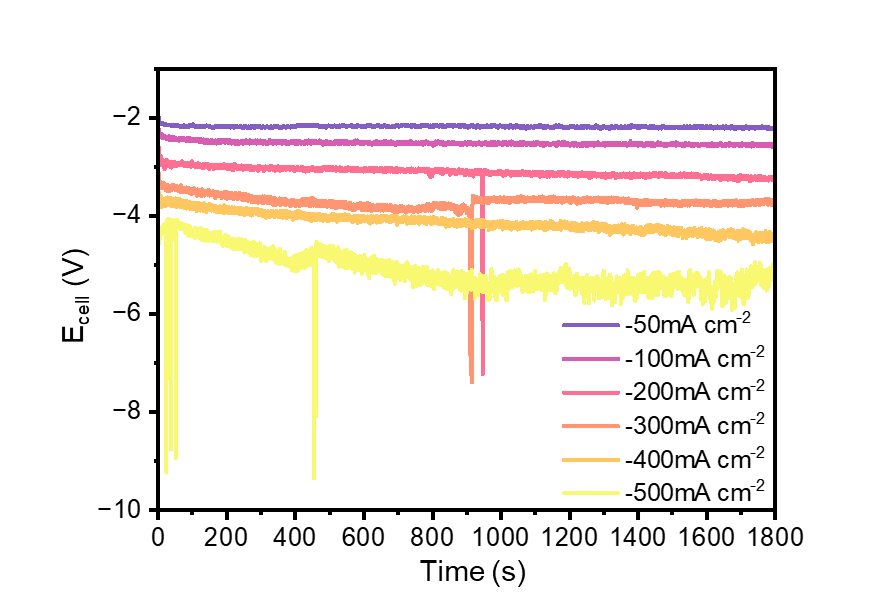


**Figure S21.** E-t curves of MPCC-Cu under different current densities during MEA testing. The large fluctuation in the middle of the curve may be caused by the generation of carbonates that cannot be discharged in a timely manner.

The fluctuations observed in Figure S21 arise from manual operational variations inherent to our laboratory-scale online GC measurements. In comparison, the stability tests performed by an external certified facility using automated systems under controlled conditions (constant temperature/humidity and regular calibration) demonstrate significantly reduced large fluctuations. This contrast highlights how standardized testing protocols with specialized equipment improve measurement consistency.


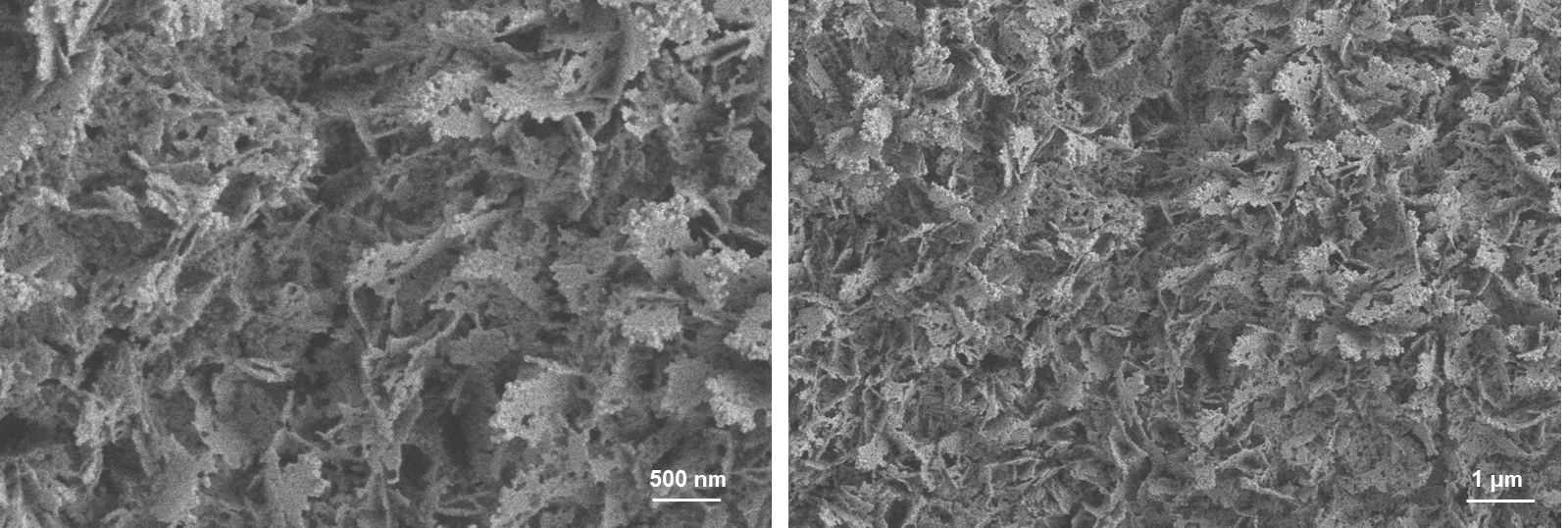


**Figure S22.** SEM images of MPCC-Cu after stability test.

**
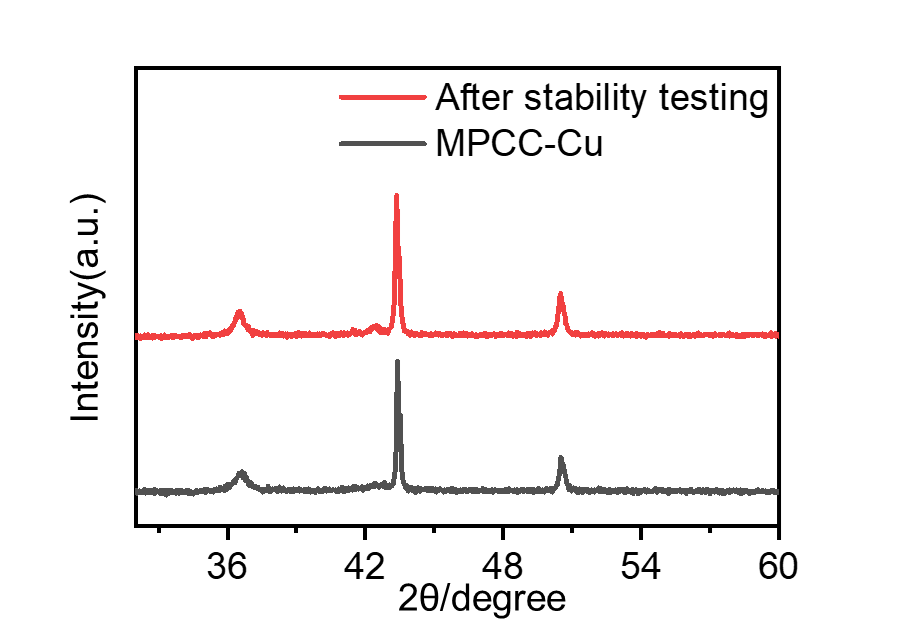
**

**Figure S23.** XRD analysis of MPCC-Cu after stability testing.


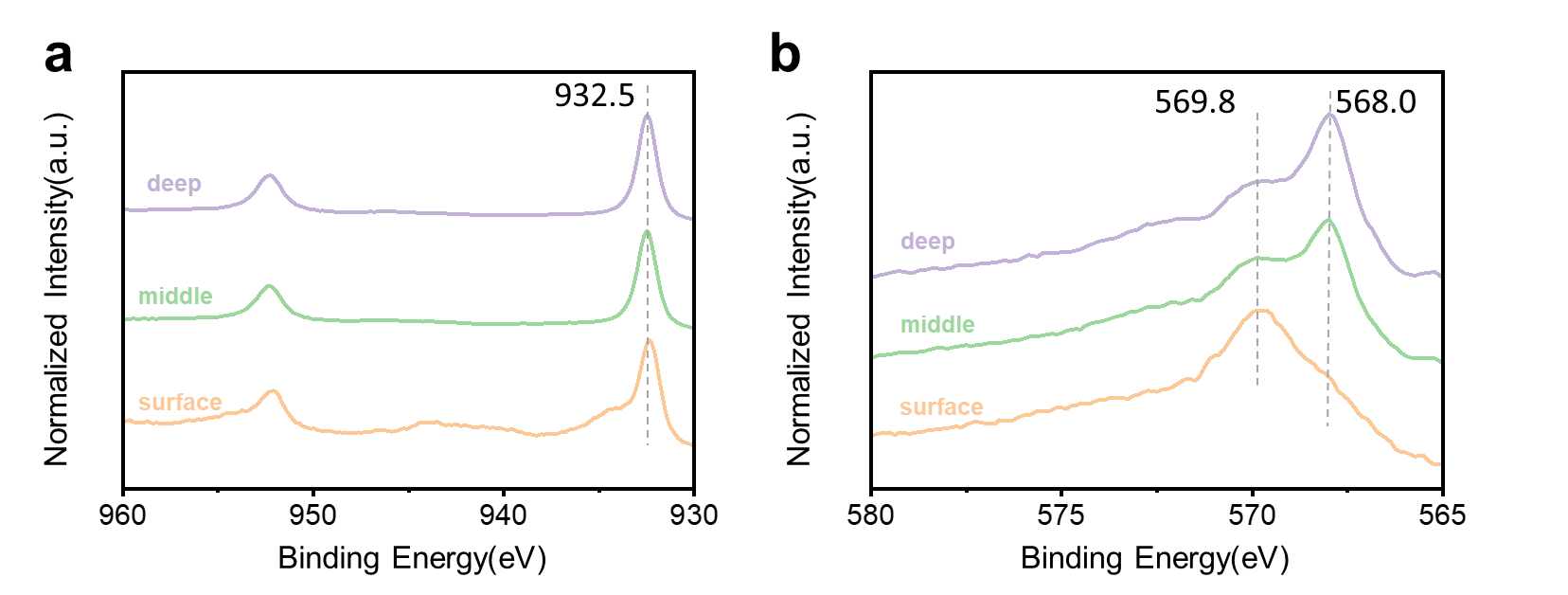


**Figure S24.** XPS spectra of (a) Cu 2p core level and (b) Cu LMM core level at different depths after 170 h stability testing of MPCC-Cu.


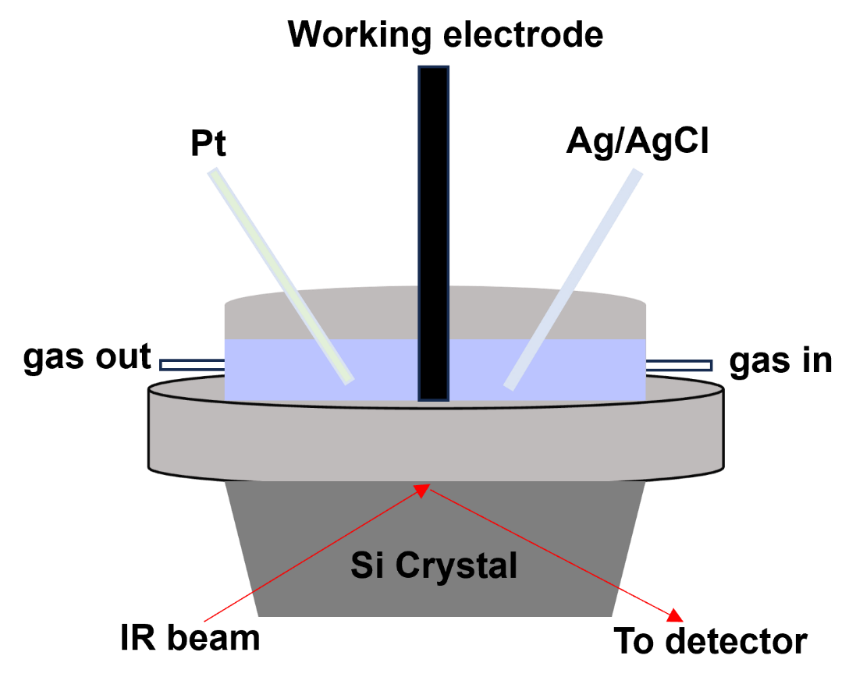


**Figure S25.** Schematic illustration of the self-made cell used for *in-situ* ATR-FTIR measurements.


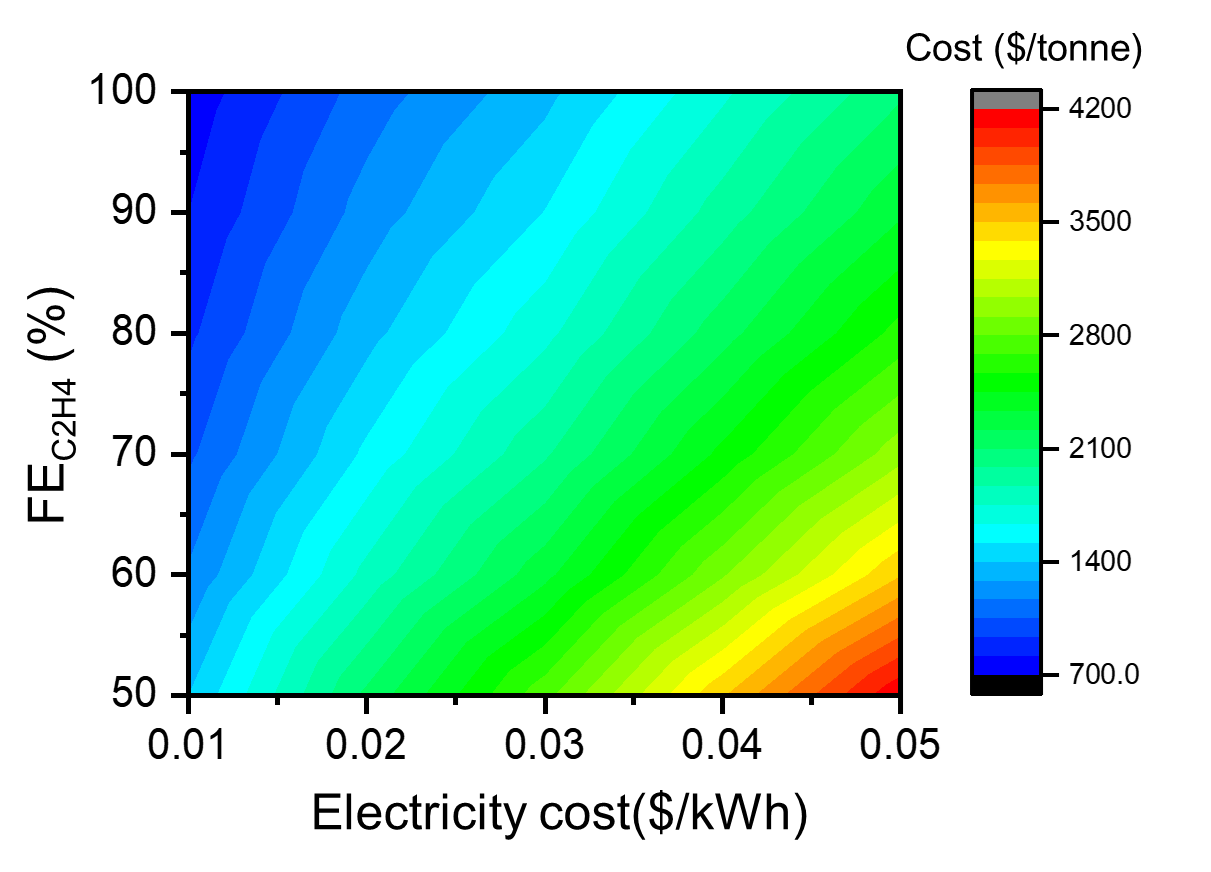


**Figure S26.** Cost analysis of C_2_H_4_ generated by CO_2_RR with changes in electricity price and FE_C2H4_.

3. Supplementary Table

**Supplementary Table S1.** Average value of FE_C2H4_.

| Time (h) | FE_C2H4_ (%) |
| --- | --- |
| 0.5 | 95.7 |
| 2 | 96.1 |
| 4 | 96.7 |
| 6 | 94.6 |
| 12 | 95.4 |
| 18 | 96 |
| 24 | 94.4 |
| 30 | 95.1 |
| 36 | 96.2 |
| 42 | 95.2 |
| 48 | 94.6 |
| 54 | 97.1 |
| 60 | 94.9 |
| 66 | 95.7 |
| 72 | 95.8 |
| 78 | 96.1 |
| 84 | 95.5 |
| 90 | 96.1 |
| 96 | 94.8 |
| 102 | 95.8 |
| 108 | 94.4 |
| 114 | 96.4 |
| 120 | 96.5 |
| 126 | 94.6 |
| 132 | 96.2 |
| 138 | 95.7 |
| 144 | 94.9 |
| 150 | 95.5 |
| 156 | 95.2 |
| 162 | 94.3 |
| 164 | 94.6 |
| 166 | 95.7 |
| 167 | 91.9 |
| 168 | 93.4 |
| 169 | 88.5 |
| 170 | 86.2 |
| average | 94.88333333 |

**Supplementary Table S2.** EXAFS fitting parameters at the Cu K-edge. (S_0_^2^=0.88)

| **Sample** | **Path** | **CN** | **R (Å)** | **σ^2^×10^3^ (Å^2^)** | **∆E (eV)** | **R-factor** |
| --- | --- | --- | --- | --- | --- | --- |
| Cu foil | Cu-Cu | 12* | 2.53±0.02 | 8.8±0.4 | 4.5±0.6 | 0.002 |
| CuO | Cu-O | 4.4±0.5 | 1.96±0.01 | 5.1±1.0 | -1.6±1.6 | 0.009 |
|  | Cu-Cu | 3.3±1.5 | 2.94±0.03 | 9.0±3.2 |  |  |
| Cu_2_O | Cu-O | 1.7±0.4 | 1.84±0.01 | 2.8±2.1 | 5.6±2.8 | 0.020 |
|  | Cu-Cu | 10.4±6.0 | 3.00 | 20.4±5.9 |  |  |
| CC-Cu | Cu-O | 2.2±0.4 | 1.94±0.01 | 1.6±1.4 | 6.8±1.9 | 0.020 |
|  | Cu-Cu | 1.7±1.5 | 2.88±0.02 | 7.9±7.4 |  |  |
| MPCC-Cu | Cu-O | 0.7±0.1 | 1.84±0.01 | 1.6±1.0 | 8.9±1.4 | 0.013 |
|  | Cu-Cu | 6.7±2.4 | 3.00±0.04 | 25.0±4.0 |  |  |

CN: coordination numbers; R: bond distance; σ^2^: Debye-Waller factors (a measure of thermal and static disorder in absorber-scatterer distances); ΔE: the inner potential correction (the difference between the zero kinetic energy value of the sample and that of the theoretical model); R-factor: goodness of the fitting.

*Fitting with fixed parameter.

S_0_^2^ is the amplitude reduction factor. The CN of Cu foil was fixed as the nominal values to obtain S_0_^2^ (0.88±0.04). Then S_0_^2^ was fixed in the subsequent fitting. The bond lengths and disorder factors of Cu-O and Cu-Cu bonds are constrained to be the same during the fitting of Cu K-edge EXAFS spectra. The *k*^3^ weighting, d*k* =1, *k*-range of 3-12 Å^-1^ and R range of 1-3 Å were used for the fitting of Cu foil; d*k* =1, *k*-range of 3-13 Å^-1^ and R range of 1-2.65 Å were used for the fitting of CuO; d*k* =1, *k*-range of 3-11 Å^-1^ and R range of 1-2.9 Å were used for the fitting of Cu_2_O; d*k* =1, *k*-range of 1-11.5 Å^-1^ and R range of 1.1-2.65 Å were used for the fitting of CC-Cu; d*k* =1, *k*-range of 3-11.96 Å^-1^ and R range of 1-3 Å were used for the fitting of MPCC-Cu. Reasonable R-factors ensure the good fitting results.

**Supplementary Table S3.** Techno-economic model parameters.

| **Input Costs** | **Value** | **Reference** |
| --- | --- | --- |
| CO_2_ ($/tonne) | 30.00 | ^[1b, 1d]^ |
| Water ($/tonne) | 5.00 | ^[1b, 1d]^ |
| Electricity ($/kWh) | 0.01 | ^[1d]^ |
| KOH ($/tonne) | 790.00 | ^[1d]^ |
| Electrolyte Concentration (M) | 1.0 | This work |
| Target Production of Potassium C_2_H_4_ (tonne/day) | 100 | This work |
| **Reactor Performance** | --- |  |
| C_2_H_4_ Faradaic Efficiency (%) | 96 | This work |
| Current Density (mA/cm^2^) | 200 (MEA cell) | This work |
| Cell Voltage (V) | 2.8 (MEA cell) | This work |
| Single-Pass Utilization (%) | 40 | This work |
| **Plant Parameters** | --- |  |
| Reference Electrolyzer Cost ($/kW) | 300 | ^[1a]^ |
| Reference Current Density (mA/cm^2^) | 200 | ^[1c]^ |
| Balance of Plant, BOP (%) | 50 | ^[1a, 1b, 1d]^ |
| Lang Factor | 1 | ^[1a, 1b, 1d]^ |
| Capacity Factor (%) | 90 | ^[1a, 1b, 1d]^ |
| Electrolyzer Lifetime (years) | 30 | ^[1a]^ |
| Catalyst + Membrane Lifetime (years) | 5 | ^[1b, 1d]^ |
| Electrolyte Lifetime (years) | 1 | ^[1b, 1d]^ |
| Discount Rate (%) | 5 | ^[1a]^ |
| Maintenance Frequency (years^-1^) | 5 | ^[1b, 1d]^ |
| Maintenance Factor (%) | 10 | ^[1b, 1d]^ |
| Anolyte Volume (L/m^2^) | 100 | ^[1a, 1b, 1d]^ |
| **Gas Separation** | --- |  |
| PSA Operational Cost (kWh/m^3^) | 0.25 | ^[1b, 1d]^ |
| PSA Reference Capital Cost ($) | 1 989 043.00 | ^[1b, 1d]^ |
| PSA Reference Capacity (m^3^/h) | 1000 | ^[1b, 1d]^ |
| PSA Capacity Scaling Factor | 0.7 | ^[1b, 1d]^ |

**Supplementary Table S4.** Performance comparisons of electrocatalysts for CO_2_ to ethylene.

| Cathode Catalyst | Cell type | Electrolyte | ***S****electivity*  FE_C2H4_ (%) | ***O****verpotential*  (V_RHE_) | *Total Activity*  J (mA⋅cm^-2^) | ***S****tability*  (h) | Ref. |
| --- | --- | --- | --- | --- | --- | --- | --- |
| MPCC-Cu | MEA | 1.0 M KOH | 96.13 | E_cell_ = -2.8V | -200 | 168 | This work |
| Carbon/Cu/PTFE | Flow cell | 0.5 M KHCO_3_/0.5 M KCl | 70 | -0.89 | -381 | 50 | J Am Chem Soc., 2023^[5]^ |
| Cu/Ni-NAC | Flow cell | 10 M KOH | 66 | -0.5 | -100 | 10 | J Am Chem Soc., 2022^[6]^ |
| Cu–polyamine | Flow cell | 1.0 M KOH | 72 | -0.97 | -433 | - | Nat Catal., 2020^[7]^ |
| Cu-Al | Flow cell | 1.0 M KOH | 80 | -2.0 | -400 | 50 | Nature, 2020^[8]^ |
| CuAl-1 | Flow cell | 1.0 M KOH | 70.1 | -2.0 | -600 | - | Energy & Environmental Science, 2022^[9]^ |
| TA-Cu | Flow cell | 1.0 M KOH | 63.6 | -1.2 | -497.2 | 10 | Angew Chem Int Ed., 2023 ^[10]^ |
| p-Cu@m-SiO_2_ | Flow cell | 1.0 M KOH | 56 | -1.5 | -380 | 10 | J Am Chem Soc., 2024^[11]^ |
| Cu-SiO_x_ | MEA | 0.1 M KHCO_3_ | 65 | E_cell_ = -4.2V | -300 | 50 | Nat Commun., 2021^[12]^ |
| Cu | Flow cell | 1 M KHCO_3_ | 40 | -1.4 | -225 | 22 | J Am Chem Soc., 2020^[13]^ |

4. References

[1] a) A. Ozden, Y. Wang, F. Li, M. Luo, J. Sisler, A. Thevenon, A. Rosas-Hernández, T. Burdyny, Y. Lum, H. Yadegari, T. Agapie, J. C. Peters, E. H. Sargent, D. Sinton, *Joule* **2021**, 5, 706; b) J. Jin, J. Wicks, Q. Min, J. Li, Y. Hu, J. Ma, Y. Wang, Z. Jiang, Y. Xu, R. Lu, G. Si, P. Papangelakis, M. Shakouri, Q. Xiao, P. Ou, X. Wang, Z. Chen, W. Zhang, K. Yu, J. Song, X. Jiang, P. Qiu, Y. Lou, D. Wu, Y. Mao, A. Ozden, C. Wang, B. Y. Xia, X. Hu, V. P. Dravid, Y.-M. Yiu, T.-K. Sham, Z. Wang, D. Sinton, L. Mai, E. H. Sargent, Y. Pang, *Nature.* **2023**, 617, 724; c) B.-H. Zhao, F. Chen, M. Wang, C. Cheng, Y. Wu, C. Liu, Y. Yu, B. Zhang, *Nature Sustainability* **2023**, 6, 827; d) W. Fang, R. Lu, F. M. Li, C. He, D. Wu, K. Yue, Y. Mao, W. Guo, B. You, F. Song, T. Yao, Z. Wang, B. Y. Xia, *Angew Chem Int Ed.* **2024**, e202319936.

[2] X. Wang, P. Ou, A. Ozden, S.-F. Hung, J. Tam, C. M. Gabardo, J. Y. Howe, J. Sisler, K. Bertens, F. P. García de Arquer, R. K. Miao, C. P. O’Brien, Z. Wang, J. Abed, A. S. Rasouli, M. Sun, A. H. Ip, D. Sinton, E. H. Sargent, *Nat Energy.* **2022**, 7, 170.

[3] Q. Yang, P. Z. Sun, L. Fumagalli, Y. V. Stebunov, S. J. Haigh, Z. W. Zhou, I. V. Grigorieva, F. C. Wang, A. K. Geim, *Nature.* **2020**, 588, 250.

[4] J. Hu, H. Zhang, W. Xu, Z. Yuan, X. Li, *Journal of Membrane Science* **2018**, 566, 8.

[5] Z. Wang, Y. Li, X. Zhao, S. Chen, Q. Nian, X. Luo, J. Fan, D. Ruan, B. Q. Xiong, X. Ren, *J Am Chem Soc* **2023**, 145, 6339.

[6] Z. Yin, J. Yu, Z. Xie, S. W. Yu, L. Zhang, T. Akauola, J. G. Chen, W. Huang, L. Qi, S. Zhang, *J Am Chem Soc* **2022**, 144, 20931.

[7] X. Chen, J. Chen, N. M. Alghoraibi, D. A. Henckel, R. Zhang, U. O. Nwabara, K. E. Madsen, P. J. A. Kenis, S. C. Zimmerman, A. A. Gewirth, *Nat Catal.* **2020**, 4, 20.

[8] M. Zhong, K. Tran, Y. Min, C. Wang, Z. Wang, C. T. Dinh, P. De Luna, Z. Yu, A. S. Rasouli, P. Brodersen, S. Sun, O. Voznyy, C. S. Tan, M. Askerka, F. Che, M. Liu, A. Seifitokaldani, Y. Pang, S. C. Lo, A. Ip, Z. Ulissi, E. H. Sargent, *Nature.* **2020**, 581, 178.

[9] S. Sultan, H. Lee, S. Park, M. M. Kim, A. Yoon, H. Choi, T.-H. Kong, Y.-J. Koe, H.-S. Oh, Z. Lee, H. Kim, W. Kim, Y. Kwon, *Energy Environ Sci.* **2022**, 15, 2397.

[10] S. Chen, C. Ye, Z. Wang, P. Li, W. Jiang, Z. Zhuang, J. Zhu, X. Zheng, S. Zaman, H. Ou, L. Lv, L. Tan, Y. Su, J. Ouyang, D. Wang, *Angew Chem Int Ed.* **2023**, 62, e202315621.

[11] W. F. Xiong, D. H. Si, H. F. Li, X. Song, T. Wang, Y. B. Huang, T. F. Liu, T. Zhang, R. Cao, *J Am Chem Soc.* **2024**, 146, 289.

[12] J. Li, A. Ozden, M. Wan, Y. Hu, F. Li, Y. Wang, R. R. Zamani, D. Ren, Z. Wang, Y. Xu, D.-H. Nam, J. Wicks, B. Chen, X. Wang, M. Luo, M. Graetzel, F. Che, E. H. Sargent, D. Sinton, *Nat Commun.* **2021**, 12, 2808.

[13] X. Wang, A. Xu, F. Li, S. F. Hung, D. H. Nam, C. M. Gabardo, Z. Wang, Y. Xu, A. Ozden, A. S. Rasouli, A. H. Ip, D. Sinton, E. H. Sargent, *J Am Chem Soc.* **2020**, 142, 3525.
